# Endothelial activation of caspase-9 promotes neurovascular injury in retinal vein occlusion

**DOI:** 10.1038/s41467-020-16902-5

**Published:** 2020-06-23

**Authors:** Maria I. Avrutsky, Crystal Colón Ortiz, Kendra V. Johnson, Anna M. Potenski, Claire W. Chen, Jacqueline M. Lawson, Alexandra J. White, Stephanie K. Yuen, Fatima N. Morales, Elisa Canepa, Scott Snipas, Guy S. Salvesen, Ying Y. Jean, Carol M. Troy

**Affiliations:** 1 Department of Pathology & Cell Biology; Vagelos College of Physicians and Surgeons, Columbia University, New York, NY 10032 USA; 2 Department of Pharmacology; Vagelos College of Physicians and Surgeons, Columbia University, New York, NY 10032 USA; 3 NCI-designated Cancer Center, Sanford Burnham Prebys Medical Discovery Institute La Jolla, La Jolla, CA 92037 USA; 4 Department of Neurology; Vagelos College of Physicians and Surgeons, Columbia University, New York, NY 10032 USA; 5 The Taub Institute for Research on Alzheimer’s Disease and the Aging Brain; Vagelos College of Physicians and Surgeons, Columbia University, New York, NY 10032 USA

**Keywords:** Cell biology, Cell death, Mechanisms of disease, Neuroscience, Pathogenesis

## Abstract

Central nervous system ischemic injury features neuronal dysfunction, inflammation and breakdown of vascular integrity. Here we show that activation of endothelial caspase-9 after hypoxia-ischemia is a critical event in subsequent dysfunction of the blood-retina barrier, using a panel of interrelated ophthalmic in vivo imaging measures in a mouse model of retinal vein occlusion (RVO). Rapid nonapoptotic activation of caspase-9 and its downstream effector caspase-7 in endothelial cells promotes capillary ischemia and retinal neurodegeneration. Topical eye-drop delivery of a highly selective caspase-9 inhibitor provides morphological and functional retinal protection. Inducible endothelial-specific caspase-9 deletion phenocopies this protection, with attenuated retinal edema, reduced inflammation and preserved neuroretinal morphology and function following RVO. These results reveal a non-apoptotic function of endothelial caspase-9 which regulates blood-retina barrier integrity and neuronal survival, and identify caspase-9 as a therapeutic target in neurovascular disease.

## Abstract (web summary)

Retinal vein occlusion can cause blindness, and features neuronal dysfunction, inflammation and breakdown of vascular integrity. Here the authors report a non-apoptotic role of endothelial caspase-9 in regulating blood-retina barrier integrity and neuronal survival, which can be therapeutically targeted in a mouse model of retinal vein occlusion.

## Introduction

The central nervous system (CNS) is one of the most metabolically active tissues in the body, rendering it exquisitely sensitive to vascular dysfunction; decreases in blood flow lead to hypoxia and ischemia which induce both neuronal loss and an increase in vascular permeability. Breakdown in endothelial barrier function allows fluid leakage into the parenchyma, leading to edema—a devastating and potentially lethal consequence in the CNS^1^. Dysregulation of the barrier has been implicated in aging-related cognitive decline^2^ and in acute and chronic neurologic disorders, including stroke, multiple sclerosis, Alzheimer’s disease, and diseases that disrupt retinal blood supply such as retinal vein occlusion (RVO) and diabetic macular edema^3^.

RVO affects between 1 and 2% of persons over the age of 40 and is the second leading cause of new blindness in working age adults^4^. Obstruction of blood flow stimulates increased expression of vascular endothelial growth factor (VEGF)^5,6^, a key component of retinal hypoxia response. Müller cells secrete VEGF^7^, which acts on endothelial cells to promote vasodilation and increase vascular permeability^8^. In patients with RVO, the initial retinal occlusions may recanalize by 2 weeks^9^, while edema and microvascular dysfunction can persist for months or years. Current treatment strategies focus on attenuating retinal edema either by vascular stabilization via anti-VEGF agents or broad-spectrum suppression of inflammation via corticosteroids^4^. The pivotal studies on anti-VEGF treatment in RVO (BRAVO and CRUISE trials) demonstrated significant reduction of retinal swelling and improved visual acuity in eyes receiving intravitreal ranibizumab, a VEGF-neutralizing antibody^10,11^. However, levels of intraocular VEGF vary widely across eyes with RVO^12^, and many treated patients continue to experience vision decline^13^. In follow-up studies of patients receiving anti-VEGF treatment, refractory edema persisted in 50% of branch RVO eyes and in 56% of central RVO eyes^14^, which suggests that additional, non-VEGF-mediated, pathways contribute to retinal edema and vision loss in RVO. Furthermore, existing interventions do not address the extensive retinal degeneration which occurs even when edema has successfully been treated^15^, highlighting the need to identify signaling pathways which can promote neuronal survival in retinal disease.

In hypoxia–ischemia it has been proposed that neuronal injury is a consequence of a hypoxia-induced cascade of pathologic signaling, including excitotoxicity, release of free radicals, and mitochondrial dysfunction^16^. The caspase family of proteases are activated in neurodegeneration, and hypoxia–ischemia increases expression of total and cleaved caspase-9 in CNS tissues^17,18^. Neuronal apoptosis in the ischemic brain is mediated by the caspase-9-dependent intrinsic (mitochondrial) death pathway, and inhibition of caspase signaling improves functional outcomes in animal models of cerebral ischemia^18–21^. In contrast to the well-studied role of caspases in neuronal death, less is known about caspase signaling in ischemic vasculature and studies have offered conflicting conclusions regarding endothelial cell death in retinal hypoxia–ischemia injury^22,23^.

The neuroretina is an accessible model system for studying barrier function in a physiologic setting^24,25^. Laser-induced RVO offers a robust model of neuronal dysfunction and loss of vascular integrity in the CNS^5,26^, and recent studies have demonstrated that mouse RVO is tractable for examining pathogenic mechanisms of hypoxia–ischemia via pharmacological approaches^6,27–31^.

Here, we report that endothelial caspase-9 activity creates a mechanistic link between vascular and neuronal injury in mouse RVO. Topical (eye-drop) application of a highly specific caspase-9 inhibitor reduces retinal ischemia and provides robust morphologic, cellular, and functional neuroprotection; genetic deletion of caspase-9 from endothelial cells phenocopies the protective effects of pharmacological caspase-9 inhibition, identifying specifically endothelial caspase-9 activity as the proximal event in hypoxia/ischemia-induced neuronal injury. In contrast to the canonical role of caspase-9 as an initiator of apoptosis, RVO-induced activation of endothelial caspase-9 is not associated with endothelial cell death. The unexpected link of endothelial caspase signaling to neuronal function provides insights into neurovascular biology and therapeutic approaches for treatment of neurovascular disease.

## Results

### Mouse RVO models hypoxic–ischemic neurovascular damage

**Fig. 1:**
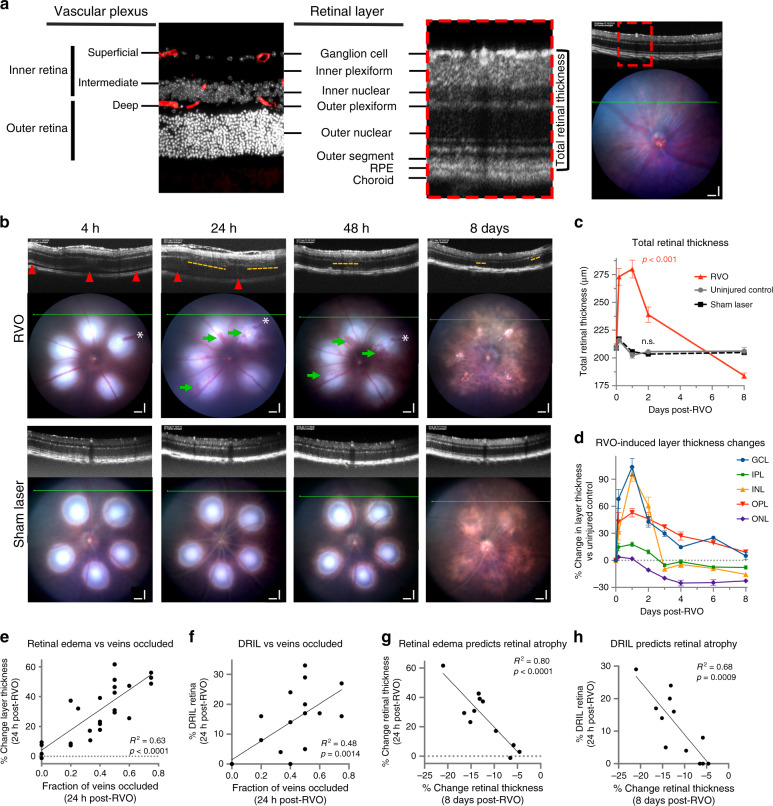
In vivo ophthalmic imaging measures pathological changes following laser-induced RVO. **a** Comparative representation of retinal layer segmentation and vascular plexi as observed by immunostaining (left; red = isolectin, white = DAPI, *n* = 6) and optical coherence tomography (OCT, *n* = 18) in vivo imaging (right) in uninjured adult C57/Bl6J mouse. Scale bar = 100 μm. **b** OCT and fundus retinal imaging at 4 h, 24 h, and 48 h, and 8 days post-RVO (*n* = 10, 21, 14, 16) or sham laser (*n* = 14). Retinal detachment (red arrowheads) and DRIL (yellow dashes) indicated on OCT imaging, and vein occlusions (white asterisks (*)) and retinal hemorrhages (green arrows) marked on fundus imaging. Scale bar = 100 μm. **c** Quantification of total retinal thickness in uninjured controls (*n* = 6), sham laser (*n* = 14), and RVO + Pen1 treated eyes (*n* = 10, 21, 14, 16) at 4 h, 24 h, 48 h, and 8 days. One-way ANOVA with Fisher’s LSD performed for each time-point; mean ± SEM. **d** Quantification of change in thickness of individual retinal layers (*n* = 10, 21, 14, 13, 4, 4, 16) at 4 h, 24 h, 48 h, 3 d, 4 d, 6 d, 8 d; mean ± SEM). **e** Retinal swelling is correlated to fraction of veins occluded at 24 h post-RVO (linear regression, *n* = 25). **f** DRIL is correlated to the fraction of veins occluded at 24 h post-RVO (linear regression, *n* = 25). **g** Retinal swelling 24 h post-RVO is correlated to retinal thinning 8 days post-RVO (linear regression, *n* = 25). **h** DRIL 24 h post-RVO is correlated to retinal thinning 8 days post-RVO (linear regression, *n* = 25). Source data are provided as a Source Data file.

To study mechanisms regulating neurovascular damage we employed a mouse RVO model of hypoxia–ischemia injury. We applied clinical in vivo imaging techniques to correlate the mouse model with the human disease. Optical coherence tomography (OCT) provides real-time optical histological cross-sections of the retina, revealing the temporal progression of edema and cell loss through distinct neuroretinal layers (Fig. 1a, Supplementary Fig. 1A). Following induction of RVO by laser photocoagulation, we observed retinal pathology consistent with ophthalmological findings in clinical RVO^4^, such as retinal detachment, retinal swelling, intraretinal hemorrhaging, and disorganization of retinal inner layers (DRIL) (Fig. 1b, c). DRIL is a robust clinical measure of capillary ischemia^32,33^, that is predictive of visual acuity loss in RVO^34–37^ and other retinopathies^38,39^.

Retinal vasculature supplies blood flow to the outer plexiform layer (OPL), and all layers of the inner retina: the ganglion cell layer (GCL), the inner plexiform layer (IPL), and the inner nuclear layer (INL) (Fig. 1a). Retinal swelling following RVO induction is driven primarily by edema in the GCL, INL, and OPL, which peaks 24 h post-RVO and subsides over 3–8 days (Fig. 1d). The photoreceptor/outer nuclear layer (ONL) is avascular and not subject to vasogenic edema. However, retinal detachment following RVO uncouples photoreceptors from the choroidal circulation, resulting in ischemia and photoreceptor degeneration^40^; retinal ischemic injury induces neuronal death in both the inner and outer retina^23^. In the mouse RVO model, retinal atrophy is notable in the photoreceptor layer by 48 h, and in the IPL and INL by 3 days post-RVO (Fig. 1d).

### Laser-induced RVO pathology correlates with the degree of RVO

A potential confounding feature of the laser-induced RVO model is direct retinal injury from the laser burns, which may trigger an inflammatory and tissue damage response^41^. To minimize this influence on our study, we limited the laser intensity and number of laser applications per vessel, and we measured retinal injury distal from the burn sites. Both edema and DRIL are correlated to the fraction of retinal veins occluded at 24 h post-RVO (Fig. 1e, f, Supplementary Fig. 1C), and both measures are highly predictive of retinal atrophy at 8 days (Fig. 1g, h). Conversely, there is no correlation between retinal edema and number of veins irradiated (Supplementary Fig. 1B).

Sham laser treatment applied to the capillary bed between major retinal veins does not induce RVO pathology (Fig. 1b, c). No instances of hemorrhaging or DRIL were observed in the sham laser treatment group.

### RVO induces caspase activation

**Fig. 2:**
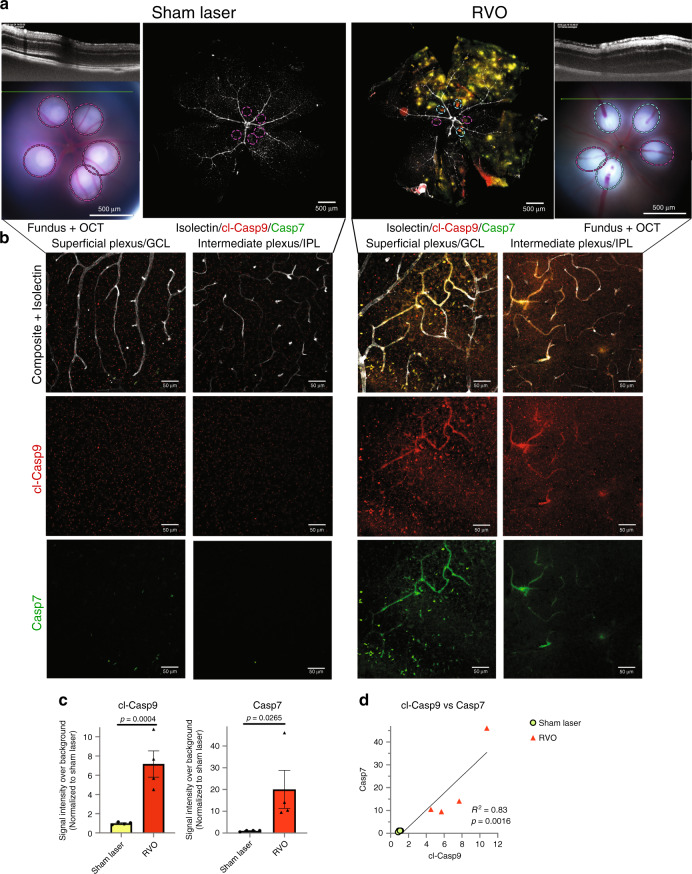
RVO induces rapid activation of caspase-9 and caspase-7. **a** In vivo imaging and immunostaining of retinas 4 h post-RVO or sham laser (white = isolectin, red = cl-caspase-9, green = caspase-7, *n* = 4, scale bar = 500 μm, burn sites indicated to scale on fundus and IHC imaging; teal oval = laser burn + occlusion, magenta oval = laser burn/no occlusion). **b** Closeups of immunostaining in (**a**), at sites distal to the location of laser application (scale bar = 50 μm). **c** Quantification of cl-caspase-9 and caspase-7 signal intensity in (**a**); two-tailed Welch’s *t* test; mean ± SEM. **d** Correlation of cl-caspase-9 and caspase-7 signal intensity in (**a**) (linear regression). cl-casp9, cl-caspase-9; casp7, caspase-7. Source data are provided as a Source Data file.

To characterize potential molecular drivers of neuronal injury in the RVO model we investigated caspase activation in retinas subjected to RVO or to sham laser. Retinal flatmounts were harvested 4 h post-RVO and immunostained with a neoepitope specific antibody to autocleaved caspase-9 (cl-caspase-9) and an antibody generated against active caspase-7. We identified induction of cl-caspase-9 and its target caspase-7 in the GCL and in the capillary bed of occluded veins within 4 h of RVO (Fig. 2, Supplementary Fig. 2). The induction of cl-caspase-9 and caspase-7 are significantly correlated (Fig. 2c, d), consistent with the role of caspase-9 as an activator of caspase-7^42^.

Neither sham laser treatment, nor application of laser burns to retinal veins without occlusion, elicits caspase activation (Fig. 2, Supplementary Fig. 2).

### RVO-induced caspase-9 is associated with neuronal, but not endothelial, cell death

**Fig. 3:**
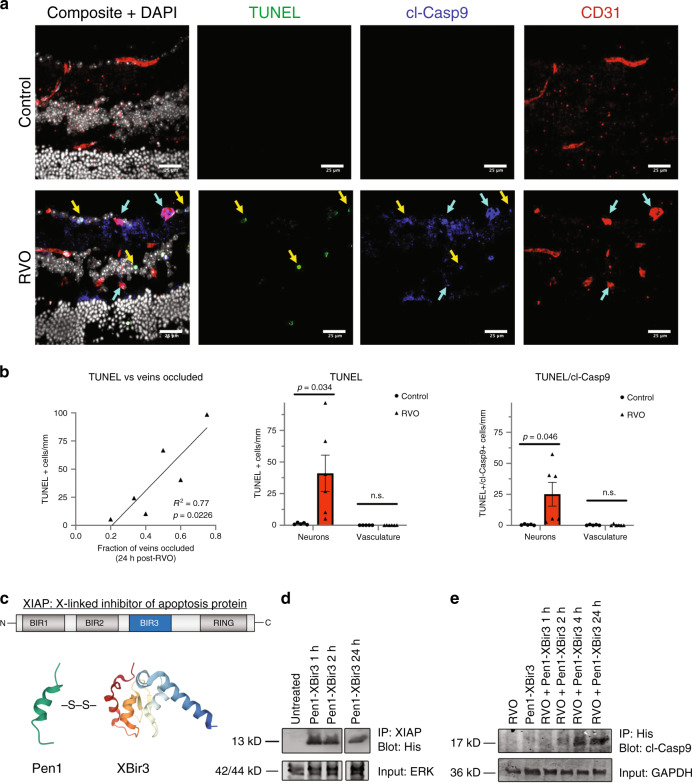
RVO-induced activation of caspase-9 in endothelial cells is nonapoptotic. **a** Retinal cross-sections of Pen1-treated control eyes (*n* = 5) and Pen1-treated eyes 24 h post-RVO (*n* = 6) stained for TUNEL (green), cl-caspase-9 (blue), isolectin (red), and DAPI (white). Yellow arrows indicate neuronal cells that are positive for TUNEL and cl-caspase-9. Teal arrows indicate vessels that are positive for cl-caspase-9. **b** Quantification of (**a**): induction of TUNEL is correlated with the fraction of veins occluded 24 h post-RVO (linear regression), RVO induces increased expression of TUNEL, and increased coexpression of TUNEL/cl-caspase-9 in neuronal nuclei and not in endothelial cells (vasculature); two-tailed Welch’s *t* test; mean ± SEM. **c** The Bir3 domain of XIAP (X-linked inhibitor of apoptosis protein) is a highly selective endogenous inhibitor of cl-caspase-9. Pen1-XBir3 is generated by crosslinking XBir3 with Penetratin-1, a cell-penetrating peptide. XBir3 and Penetratin-1 structure rendered with Mol*^70^. **d** Western blot showing detection of XBir3 in uninjured retinal lysates at 1, 2, and 24 h following administration of Pen1-XBir3 eye-drops in mice. *n* = 3. **e** Western blot of target engagement; mice received Pen1-XBir3 eye-drops immediately following induction of RVO. Immunoprecipitation of XBir3 from retinal lysates shows XBir3 binding to cl-caspase-9. *n* = 2. cl-casp9, cl-caspase-9. Source data are provided as a Source Data file.

To determine if RVO-induced activation of caspase-9 was associated with cell death, we utilized terminal deoxynucleotidyl transferase dUTP nick end labeling (TUNEL) to mark the nuclei of dying cells, and evaluated the colocalization of TUNEL and cl-caspase-9 in neuronal cell layers and in endothelial cells (Fig. 3a, b). The induction of TUNEL correlates with the fraction of veins occluded at 24 h post-RVO. RVO induces a significant increase in TUNEL, as well as in colocalization of cl-caspase-9 and TUNEL in neuronal cells, but not in the vasculature (Fig. 3a, b), indicating that RVO induces cell-type specific regulation of caspase-9 activity, and that caspase-9 is not causing death in endothelial cells.

### Retinal delivery of Penetratin-1-linked XBir3 via topical eye-drops inhibits caspase-9

To interrogate the function of cl-caspase-9 in RVO, we utilized Pen1-XBir3, a cell penetrating highly selective caspase-9 inhibitor^18,43^, comprised of Penetratin-1 (a cell-penetrating peptide) cross-linked to XBir3 (the caspase-9 inhibiting domain of XIAP protein) (Fig. 3c). Penetratin-1 is an absorption enhancer for noninvasive intraocular drug delivery^44^. To test if topical application of Pen1-XBir3 would deliver XBir3 into retinal tissues, mice were treated with eye-drops containing 10 μg Pen1-XBir3, and retinas were harvested for analysis at the indicated time-points (Fig. 3d). Retinal lysates were immunoprecipitated with an XIAP antibody generated to the Bir3 domain, followed by western blot for His (the XBir3 construct contains a His-tag). XBir3 is detected in retinal lysates at 1 h post-treatment, and is still detectable in the retina at 24 h post-treatment (Fig. 3d).

Previous studies demonstrate that XBir3 targets auto-cleaved caspase-9^45^. To assess target engagement in the retina, mice were subjected to RVO, with or without administration of Pen1-XBir3 eye-drops immediately after occlusion. Retinas were harvested at the indicated times for analysis: immunoprecipitation of His, followed by western blot for caspase-9. XBir3 and its target caspase-9 co-precipitate by 2 h post-RVO, and target engagement is still evident at 24 h post-RVO (Fig. 3e).

To determine if topical delivery of Pen1-XBir3 is scalable to a larger eye, we tested retinal uptake of Pen1-XBir3 eye-drops in rabbits. Pen1-XBir3 eye-drops were administered twice-daily to rabbits for 5 consecutive days. We detected XBir3 in retinal lysates, but not in the blood plasma of treated rabbits (Supplementary Fig. 3) supporting local delivery of XBir3 to the retina.

### Pen1-XBir3 eye-drops selectively inhibit caspase-9 activity

**Fig. 4:**
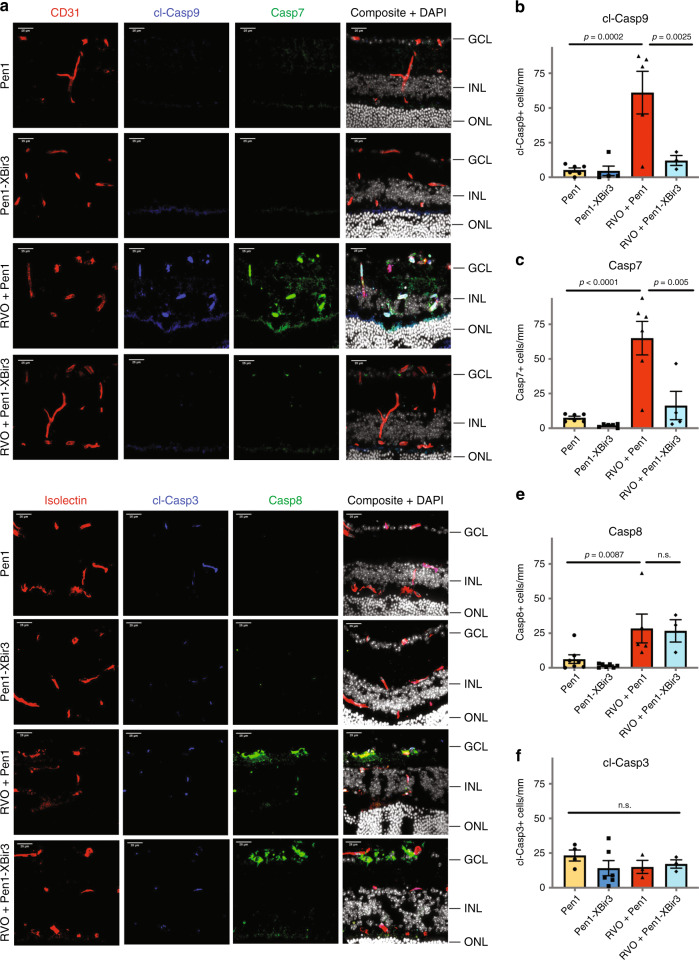
Pen1-XBir3 eye-drops inhibit Caspase-9 activity after RVO. **a** Retinal cross-sections from control eyes and 24 h post-RVO, treated with Pen1 or Pen1-XBir3 and immunostained for CD31 (red), cl-caspase-9 (blue), caspase-7 (green), and DAPI (white) (scale bar = 25 μm). **b** Quantification of cl-caspase-9; one-way ANOVA with Fisher’s LSD, mean ± SEM. Pen1 (*n* = 6), Pen1-XBir3 (*n* = 5), RVO + Pen1 (*n* = 5), RVO + Pen1-XBir3 (*n* = 3). **c** Quantification of caspase-7; one-way ANOVA with Fisher’s LSD, mean ± SEM. Pen1 (*n* = 6), Pen1-XBir3 (*n* = 6), RVO + Pen1 (*n* = 6), RVO + Pen1-XBir3 (*n* = 4). **d** Retinal cross-sections from control eyes and 24 h post-RVO, treated with Pen1 or Pen1-XBir3 and immunostained for isolectin (red), cl-caspase-3, caspase-8 (green), and DAPI (white) (scale bar = 25μm). **e** Quantification of caspase-8; one-way ANOVA with Fisher’s LSD, mean ± SEM. Pen1 (*n* = 7), Pen1-XBir3 (*n* = 7), RVO + Pen1 (*n* = 5), RVO + Pen1-XBir3 (*n* = 3). **f** Quantification of cl-caspase-3; one-way ANOVA with Fisher’s LSD, mean ± SEM. Pen1 (*n* = 4), Pen1-XBir3 (*n* = 6), RVO + Pen1 (*n* = 3), RVO + Pen1-XBir3 (*n* = 3). cl-casp9, cl-caspase-9; casp7, caspase-7; cl-casp3, cl-caspase-3; casp8, and caspase-8. Source data are provided as a Source Data file.

To measure the effect of Pen1-XBir3 on caspase-9 activity and downstream signaling pathways in retinal tissues, we immunostained retinal cross-sections for cl-caspase-9 and caspase-7 (Fig. 4a). Isolectin and CD31 were used to identify vasculature in retinal cross-sections. In uninjured eyes, cl-caspase-9 and caspase-7 are expressed at low levels colocalizing with neuronal processes in the IPL and OPL (Fig. 4a, Supplementary Fig. 4A). At 24 h post-RVO, levels of cl-caspase-9 and caspase-7 increase dramatically in both endothelial cells and in retinal neurons (Fig. 4a). Treatment with Pen1-XBir3 eye-drops immediately after RVO blocks caspase-9 autocleavage and abrogates induction of caspase-7 (Fig. 4a–c), supporting caspase-9 as the main protease responsible for activation of caspase-7 in RVO. The induction of cl-caspase-9 and caspase-7 correlates with the fraction of veins occluded in Pen1 treated, but not in Pen1-XBir3 treated eyes (Supplementary Fig. 4B). In addition to activating caspase-7, caspase-9 can activate caspase-3 and caspase-6. We detected a moderate increase in cl-caspase-6 signal in retinal neurons and astrocytes following RVO, which is also attenuated by Pen1-XBir3 (Supplementary Fig. 4C, D). Basal levels of cl-caspase-3 colocalize with isolectin-positive cells, and are not modulated by either RVO or Pen1-XBir3 (Fig. 4d, f). RVO also induces increased retinal expression of caspase-8 and caspase-1, which are not regulated by Pen1-XBir3 (Fig. 4d, e, Supplementary Fig. 4D). Treatment with Pen1-XBir3 limits the induction of TUNEL 24 h post-RVO (Supplementary Fig. 4E), indicating that caspase-9 activity promotes retinal cell death following RVO.

### Blocking caspase-9 activity rescues vascular integrity after RVO

**Fig. 5:**
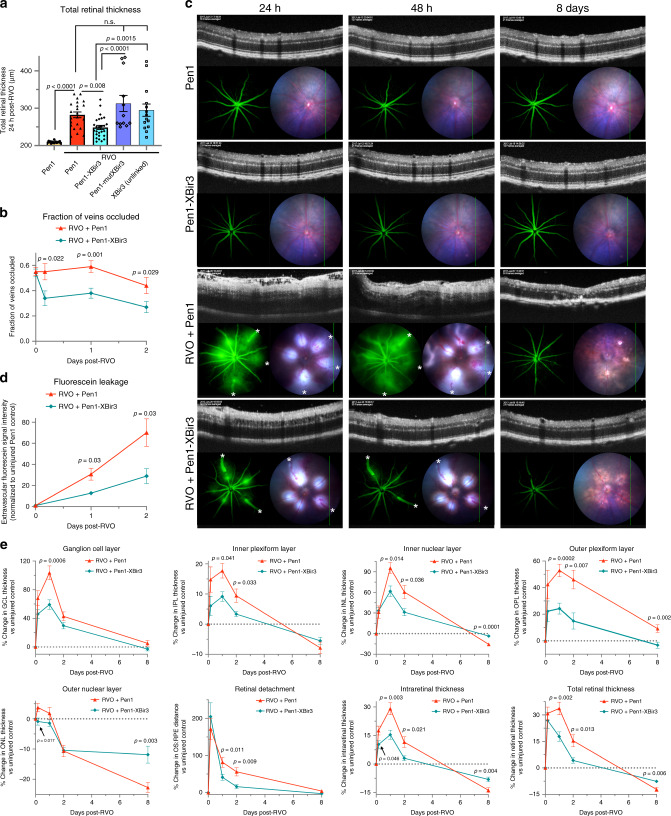
Caspase-9 inhibitor reduces edema and rescues retinal morphology after RVO. **a** OCT measure of total retinal thickness in Pen1-treated control eyes (*n* = 18), and 24 h post-RVO in eyes treated with Pen1 (*n* = 22), Pen1-XBir3 (*n* = 26), Pen1-XBir3 (inactive mutant) (*n* = 12), and XBir3 (unlinked) (*n* = 14). One-way ANOVA with Fisher’s LSD; mean ± SEM. **b** Quantification of the fraction of veins occluded immediately after induction of RVO, and at 4 h, 24 h, and 48 h post-RVO. (RVO + Pen1, *n* = 39, 19, 28, 20) (RVO + Pen1-XBir3, *n* = 52, 18, 31, 25) two-tailed Welch’s *t* test; mean ± SEM. **c** Fluorescein angiography, OCT, and fundus retinal imaging of eyes treated with Pen1 (*n* = 5) or Pen1-XBir3 (*n* = 7) following induction of RVO, imaged at 24 h, 48 h, and 8 days (white asterisks (*) = vein occlusions) and uninjured Pen1-treated (*n* = 12) and Pen1-Xbir3-treated (*n* = 12) eyes. **d** Fluorescein leakage quantification from (**c**); two tailed Welch’s *t* test; mean ± SEM. **e** Quantification of changes in average OCT retinal thickness in specific retinal layers relative to baseline thickness of uninjured controls. RVO + Pen1 (*n* = 10, 21, 14, 16), RVO + Pen1-XBir3 (*n* = 12, 26, 16, 14) at 4 h, 24 h, 48 h, and 8 days; two-tailed Welch’s *t* test; mean ± SEM. Source data are provided as a Source Data file.

To investigate if caspase-9 inhibition modulates RVO-induced hypoxia–ischemia injury, we measured retinal pathology in mice which received either Pen1-XBir3 or Pen1 eye-drops (Supplementary Fig. 5A). Mice received two doses of eye-drops (immediately post-RVO, and at 24 h post-RVO) to maintain caspase-9 inhibition throughout the edema phase of RVO injury. The location of laser burns, numbers of veins irradiated, and fraction of veins occluded was consistent between Pen1-XBir3 and Pen1-treated eyes and treatment with Pen1-XBir3 did not alter the dilation of occluded veins following RVO (Supplementary Fig. 5). Treatment with Pen1-XBir3 reduces retinal thickness 24 h post RVO, while Pen1 alone, XBir3 alone, and Pen1-mutXBir3 (an inactive mutant) do not regulate retinal edema (Fig. 5a).

Since retinal pathology is correlated with the fraction of veins occluded at 24 h post-RVO, we investigated the effect of Pen1-XBir3 treatment on the resolution of occlusions. Recanalization of occluded vessels usually occurs spontaneously within 1 week in laser-induced models of RVO^26^. In Pen1-treated eyes, vein occlusions remain mostly stable through 48 h, while treatment with Pen1-XBir3 significantly reduces the fraction of veins occluded by 4 h post-RVO (Fig. 5b), suggesting that increased vessel reperfusion may be part of the therapeutic effect of caspase-9 inhibition.

We used fluorescein, a small fluorescent dye used extensively in ophthalmology to diagnose and manage treatment of vascular disorders^4^, to assess the disruption of endothelial barrier function after RVO. Increased fluorescein leakage is present 24–48 h post-RVO, which coincides with peak retinal swelling as measured by OCT (Fig. 5c, d). Treatment with Pen1-XBir3 substantially reduces the amount of fluorescein leakage in injured eyes.

### In vivo OCT imaging measures protection of retinal morphology by caspase-9 inhibition

Mice treated with Pen1-XBir3 immediately after RVO have significantly less retinal swelling compared to Pen1-treated eyes (Fig. 5c, e). During peak edema at 24 h post-RVO, treated mice have less retinal swelling in each of the vascular layers, and less accumulation of subretinal fluid/retinal detachment. By blocking caspase-9 mediated neuronal death and reducing retinal edema, treatment with Pen1-XBir3 prevents atrophy of the INL, preserves photoreceptors in the ONL, and reduces total retinal thinning.

### Caspase-9 inhibition does not change VEGF expression

Since VEGF is a major driver of retinal edema and key target of current therapies for RVO, we investigated whether caspase-9 inhibition by Pen1-XBir3 modulated VEGF levels in the retina. VEGF levels increase in retinal lysates 24 h after RVO; treatment with Pen1-XBir3 blocks the increase in caspase-9, but does not affect VEGF levels (Supplementary Fig. 5E, F), indicating that activation of caspase-9 is an early response to RVO which occurs in parallel with induction of VEGF, and that caspase-9-mediated retinal injury is independent of VEGF expression.

### Caspase-9 inhibition attenuates hyperreflective foci

Several clinical and animal studies have proposed tracking of hyperreflective foci (HRF) on OCT imaging as a readout of retinal inflammation^46–48^. The exact etiology of HRFs remain controversial, with hypotheses suggesting lipid extravasation, migrating RPE cells, activated microglial cells and extravasated blood components as possible sources^49,50^. However, in patients with RVO, retinal HRF correlate with disease severity and predict visual outcome after treatment^51,52^.

**Fig. 6:**
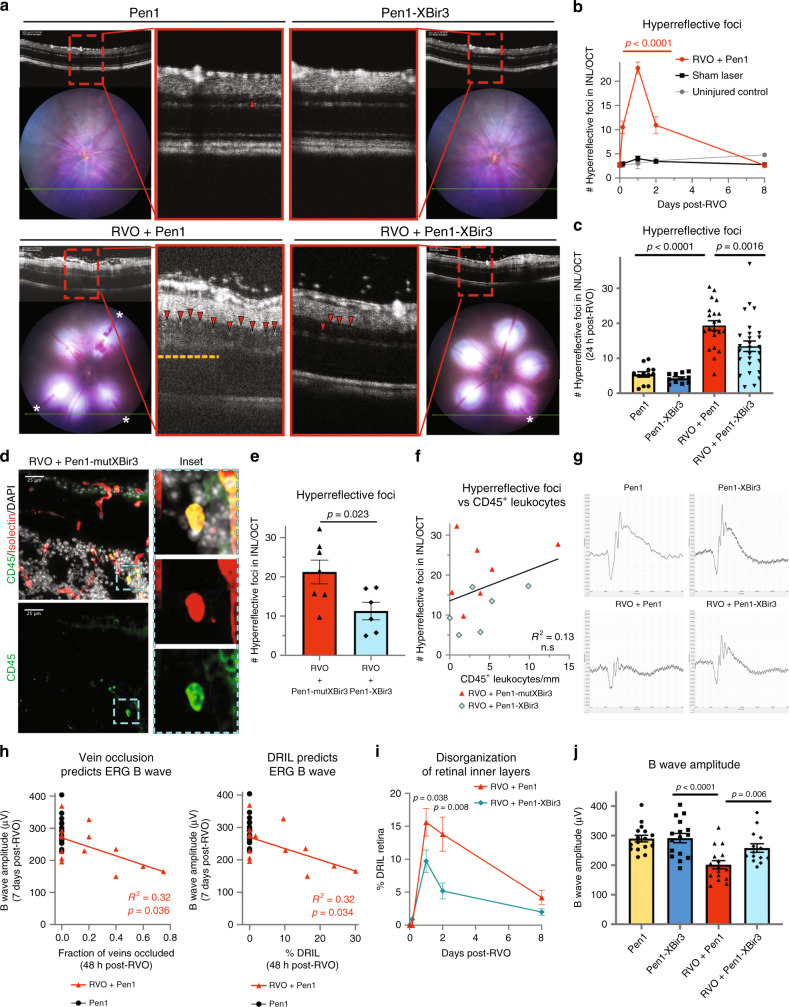
Inhibiting caspase-9 reduces hyperreflective foci and preserves retinal function. **a** Pen1-XBir3 eye-drops reduce number of hyperreflective foci (HRF) (red arrowheads) and improve DRIL (yellow dashes) 24 h post-RVO (white asterisks (*) = vein occlusions). Pen1 (*n* = 12), Pen1-XBir3 (*n* = 11), RVO + Pen1 (*n* = 21), RVO + Pen1-XBir3 (*n* = 27). **b** Quantification of HRF in uninjured controls (*n* = 6), sham laser (*n* = 14), and RVO + Pen1 treated eyes (*n* = 15, 21, 13, 16) at 4 h, 24 h, 48 h, and 8 days. One-way ANOVA with Fisher’s LSD; mean ± SEM. **c** Quantification of HRF in uninjured and 24 h post-RVO eyes treated with Pen1 or Pen1-XBir3 (Pen1 (*n* = 12), Pen1-XBir3 (*n* = 11), RVO + Pen1 (*n* = 21), RVO + Pen1-XBir3 (*n* = 27)), one-way ANOVA with Fisher’s LSD; mean ± SEM. **d** Retinal cross-section immunostained for CD45 (green), isolectin (red), and DAPI (white) (scale bar = 25 μm); Pen1-mutXBir3 (*n* = 7) at 24 h post-RVO. Inset depicts a leukocyte in the INL expressing CD45 and isolectin. **e** Quantification of HRF in OCTs from RVO + Pen1-mutXBir3 (*n* = 7) and RVO + Pen1-XBir3 (*n* = 6) at 24 h post-RVO. **f** Correlation between CD45^+^ leukocyte counts from D and HRF counts from E (linear regression). **g** Representative ERG traces 7 days post-RVO show reduced b wave amplitude in Pen1-treated eyes, rescued by treatment with Pen1-XBir3. **h** ERG b wave amplitude 7 days post-RVO is correlated to the fraction of veins occluded 48 h post-RVO, and with DRIL (linear regression, *n* = 14). **i** Quantification of DRIL in RVO-induced eyes treated with Pen1 (*n* = 11, 20, 13, 16) or Pen1-XBir3 (*n* = 12, 26, 16, 16) at 4 h, 24 h, 48 h, and 8 days. Two-tailed Welch’s *t* test; mean ± SEM. **j** Quantification of b wave amplitude in uninjured control (*n* = 16) and 7 days post-RVO in Pen1 (*n* = 16) and Pen1-XBir3 (*n* = 14) treated eyes; One-way ANOVA with Fisher’s LSD; mean ± SEM. Source data are provided as a Source Data file.

We observed induction of retinal HRF during the edema phase of RVO (Fig. 6a, b). Numbers of HRF correlate with the fraction of veins occluded and with retinal edema, and are predictive of retinal atrophy at 8 days post-RVO (Supplementary Fig. 6). Sham laser treatment does not induce increased HRF (Fig. 6c), indicating that HRF induction is caused by hypoxia–ischemia, rather than by laser injury. Pen1-XBir3 reduces the number of HRF in the retina (Fig. 6a, c).

Since inflammatory cells can present as hyperreflective dots on OCT imaging^53^, we sought to investigate if the induction of HRF were related to leukocyte infiltration. We immunostained Pen1-XBir3 and Pen1-mutXBir3-treated eyes 24 h post-RVO with CD45 (leukocyte common antigen) and isolectin (Fig. 6d). CD45^+^ leukocytes in retinal tissues correlate with retinal swelling 24 h post-RVO, but not with the fraction of veins occluded (Supplementary Fig. 6D, E). However, the number of leukocytes do not correlate with HRF on OCT imaging, ruling out leukocyte infiltration as a primary cause of retinal HRF in RVO (Fig. 6e, f).

### Electroretinography (ERG) measures Pen1-XBir3 rescue of neuroretinal function in RVO

To test whether morphologic preservation of neuronal retinal layers also preserved retinal function, we utilized scotopic focal ERG, 7 days post-RVO. The ERG b wave amplitude, which reflects post-phototransduction activity of inner retinal neurons, is highly sensitive to deficits in retinal metabolic energy such as those caused by tissue ischemia^54^. Deficits in ERG b wave amplitude reflect the degree of retinal ischemia in patients with RVO^55^, and represent a functional measure of potential therapeutic efficacy of drugs that modulate retinal ischemic damage^56^.

RVO causes a significant reduction in ERG b wave, and a modest reduction in ERG a wave (Fig. 6g), consistent with reported findings in patients^55^ and in mouse RVO^27^. The fraction of major veins occluded and DRIL at 48 h post-RVO are predictive of ERG b wave deficits at 7 days post-RVO, and laser alone is not sufficient to cause ERG impairments if ischemia fully resolves by 48 h (Fig. 6h), supporting the link between tissue ischemia and deficits in neuronal function in RVO. Treatment with Pen1-XBir3 significantly reduces the amount of DRIL post-RVO (Fig. 6i), and rescues ERG b wave amplitude (Fig. 6j, Supplementary Fig. 6G), with similar trends in the sum amplitudes of the oscillatory potentials (Fig. 6j), indicating functional preservation of the neuroretina.

### Cdh5 Cre drives inducible deletion of caspase-9 specifically in endothelial cells

Inhibition of caspase-9 with Pen1-XBir3 is not cell-specific, and would target both neuronal and endothelial caspase-9. To interrogate the cell-specific role of endothelial caspase-9 in RVO pathology, we generated an inducible endothelial caspase-9 knockout mouse (Casp9 iEC KO) by crossing *caspase-9 flox/flox* (Casp9 WT) animals^57^ with an inducible Cre line targeting an endothelial cell promoter (*Cdh5PAC-CreERT2*)^58^. Polymerase chain reaction genotyping confirms induction of *caspase-9* recombination in tissue digests from Casp9 iEC KO animals (Supplementary Fig. 7A).

Specificity and efficacy of recombination were tested by crossing the inducible endothelial Cre mice with a Tomato-EGFP mT/mG reporter mouse^59^. Recombination is induced specifically in the vasculature, as visualized by GFP staining of retinal flatmounts 2 weeks after tamoxifen treatment in 6-week-old mice (Supplementary Fig. 7B, C). We did not find any differences in baseline retinal morphology, vasculature or ERG response between Casp9 iEC KO animals and littermate controls (Supplementary Fig. 7).

### Inducible endothelial cell deletion of caspase-9 reduces neuronal injury following RVO

**Fig. 7:**
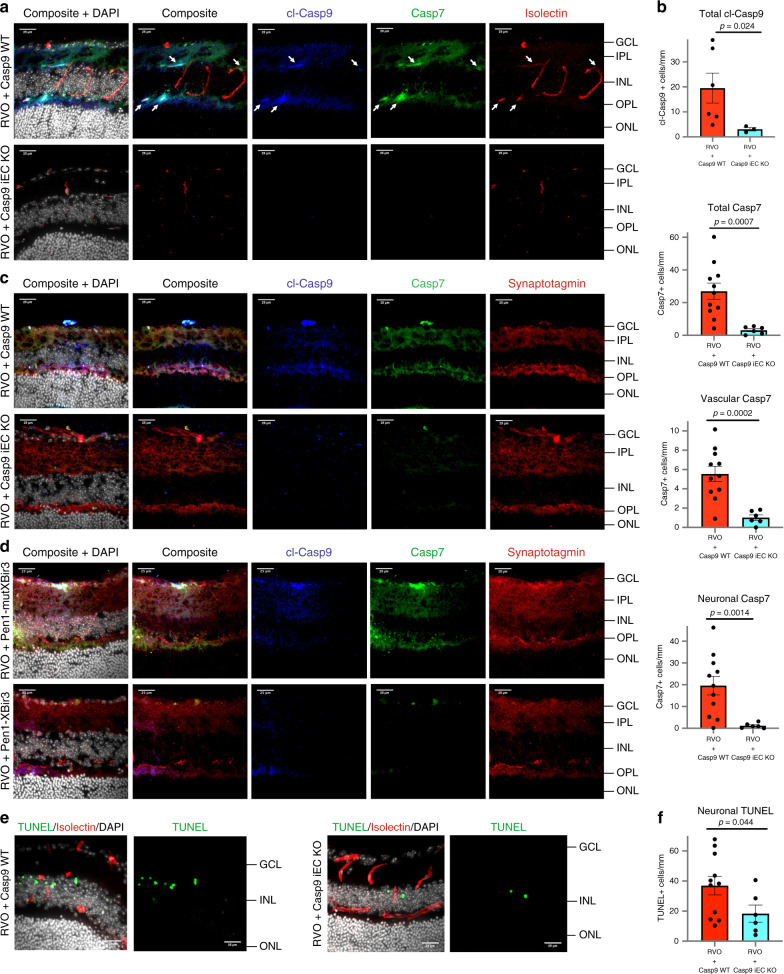
Endothelial caspase-9 mediates neuronal injury following RVO. **a** Retinal cross-sections 24 h post-RVO from Casp9 WT and Casp9 iEC KO mice immunostained for cl-caspase-9 (blue), caspase-7 (green), isolectin (red), and DAPI (white). White arrows = vessels expressing cl-caspase-9 and caspase-7. Scale bar = 25 µm. **b** Quantification of cl-caspase-9 (Casp9 iEC KO *n* = 3, Casp9 WT *n* = 6) and caspase-7 (Casp9 iEC KO *n* = 6, Casp9 WT *n* = 11) cell counts and quantification of Casp7 in neuronal vs. vascular cells 24 h post-RVO in Casp9 WT (*n* = 11) and Casp9 iEC KO eyes (*n* = 6) from (**a**); two-tailed Welch’s *t* test; mean ± SEM. **c** Retinal cross-sections 24 h post-RVO from Casp9 WT (*n* = 11) and Casp9 iEC KO (*n* = 6) eyes immunostained for cl-caspase-9 (blue), caspase-7 (green), synaptotagmin (red), and DAPI (white). Scale bar = 25 µm. **d** Retinal cross-sections 24 h post-RVO from C57Bl/6J mice treated with either (inactive) Pen1-mutXBir3 (*n* = 3) or Pen1-XBir3 (*n* = 2), immunostained for cl-caspase-9 (blue), caspase-7 (green), synaptotagmin (red), and DAPI (white). Scale bar = 25 µm. **e** Retinal cross-sections of Casp9 WT (*n* = 11) and Casp9 iEC KO (*n* = 6) eyes 24 h post-RVO, stained for TUNEL (green), isolectin (red) and DAPI. **f** Quantification of neuronal TUNEL + nuclei in Casp9 WT (*n* = 11) and Casp9 iEC KO (*n* = 6) 24 h post-RVO; two-tailed Welch’s *t* test; mean ± SEM. cl-casp9, cl-caspase-9; casp7, caspase-7. Source data are provided as a Source Data file.

Following induction of RVO in Casp9 WT mice, cl-caspase-9 and caspase-7 colocalize with isolectin in the vasculature, and with synaptotagmin in neuronal processes (Fig. 7a–c). Deletion of endothelial caspase-9 blocks the induction of cl-caspase-9 and caspase-7 throughout the retina at 24 h post-RVO. Both vascular and neuronal induction of caspase-7 are abrogated in Casp9 iEC KO mice, recapitulating the effect of non-cell specific pharmacological inhibition of caspase-9 by Pen1-XBir3 in C57Bl/6J mice (Fig. 7d). Moreover, Casp9 iEC KO mice have less neuronal TUNEL 24 h post-RVO (Fig. 7e, f), indicating that caspase-9 mediated endothelial dysfunction drives neuronal injury in RVO.

### Endothelial caspase-9 mediates vascular edema and neuronal injury

**Fig. 8:**
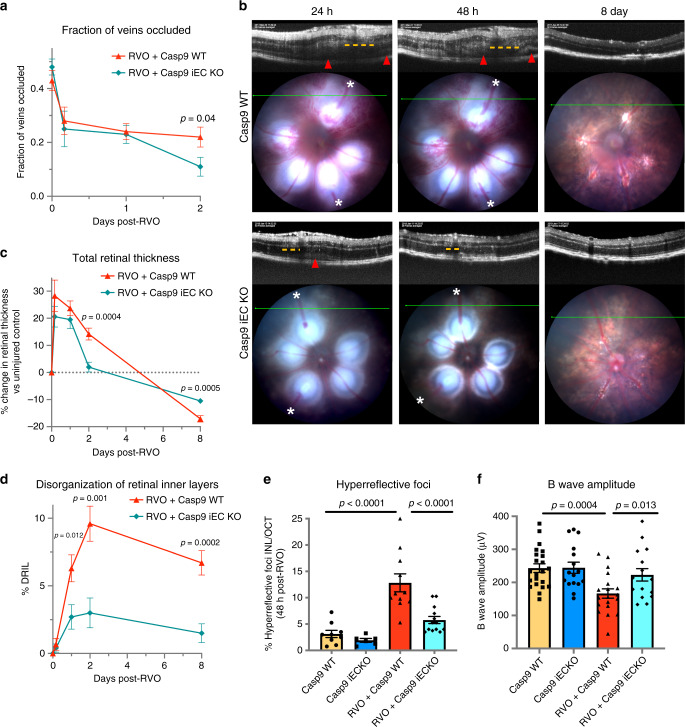
Endothelial caspase-9 contributes to retinal injury following RVO. **a** Quantification of the fraction of veins occluded immediately after induction of RVO, and at 4 h, 24 h, and 48 h post-RVO. (RVO + Casp9 WT, *n* = 50, 19, 42, 17) (RVO + Casp9 iEC KO, *n* = 50, 16, 40, 24) two-tailed Welch’s *t* test; mean ± SEM. **b** OCT and fundus retinal imaging from Casp9 WT and Casp9 iEC KO mice at 24 h, 48 h, and 8 days post-RVO. Retinal detachment (red arrowhead) and regions of DRIL (yellow dashes) indicated on OCT, vein occlusions (white asterisks (*)) indicated on fundus image. **c** Quantification of the change in total retinal thickness vs. uninjured control in Casp9 WT (*n* = 12, 22, 12, 10) and Casp9 iEC KO (*n* = 7, 19, 11, 13) eyes at 4 h, 24 h, 48 h, and 8 days post-RVO. Two-tailed Welch’s *t* test; mean ± SEM. **d** Quantification of DRIL in Casp9 WT (*n* = 12, 22, 12, 10) and Casp9 iEC KO (7, 19, 11, 13) eyes at 4 h, 24 h, 48 h, and 8 days post-RVO. Two-tailed Welch’s *t* test; mean ± SEM. **e** Quantification of HRF in uninjured and 48 h post-RVO in Casp9 WT (uninjured *n* = 9, RVO *n* = 11) and Casp9 iEC KO eyes (uninjured *n* = 6, RVO *n* = 13), one-way ANOVA with Fisher’s LSD; mean ± SEM. **f** Quantification of b wave amplitude 7 days post-RVO in Casp9 WT (uninjured *n* = 22, RVO *n* = 19) and Casp9 iEC KO eyes (uninjured *n* = 16, RVO *n* = 16) (flash intensity = 2.3 log(Cd/m^2^); one-way ANOVA with Fisher’s LSD; mean ± SEM. Source data are provided as a Source Data file.

Retinal pathology in Casp9 iEC KO mice following RVO phenocopies vascular and neuronal protection by Pen1-XBir3. Reperfusion of occluded vessels occurs faster in Casp9 WT animals compared to the C57/Bl6J strain, however by 48 h post-RVO, there is a significant reduction in occlusion rate of Casp9 iEC KO mice compared to Casp9 WT littermates (Fig. 8a). Casp9 iEC KO mice have complete resolution of retinal edema by 48 h post-RVO (Fig. 8b, c) with less retinal detachment and significant protection in all retinal layers (Supplementary Fig. 8), and less retinal atrophy at 8 days compared to Casp9 WT littermates. Reduction in DRIL (Fig. 8d) suggests that improvement of capillary nonperfusion may underlie neuronal protection in Casp9 iEC KO animals. Quantification of retinal HRF showed a 50% reduction in speck counts in Casp9 iEC KO animals (Fig. 8e). At 1 week after RVO, Casp9 WT mice have a reduction in ERG oscillatory potential amplitudes and a significant decrease in ERG b wave amplitude, which is rescued in Casp9 iEC KO animals (Fig. 8f, Supplementary Fig. 7H). These results indicate that caspase-9 signaling in endothelial cells mediates vascular edema, neuronal dysfunction and neuronal death and that targeting caspase signaling in endothelial cells can attenuate ischemic injury in CNS tissues.

## Discussion

Prior studies of CNS ischemic injury have demonstrated that hypoxia/ischemia induces temporal and spatial changes in neuronal function and vascular integrity, but it has not been elucidated whether these events are mechanistically integrated or separate pathways. RVO triggers inflammatory and hypoxic responses, both of which contribute to breakdown of the blood-retina barrier^1^. We employed an established mouse model of RVO to characterize the contribution of retinal ischemia to pathological symptoms common in retinal vascular occlusive disorders. Using clinically relevant in vivo imaging, we show close association between measures of obstructed blood flow, and subsequent functional and morphological retinal pathologies.

**Fig. 9:**
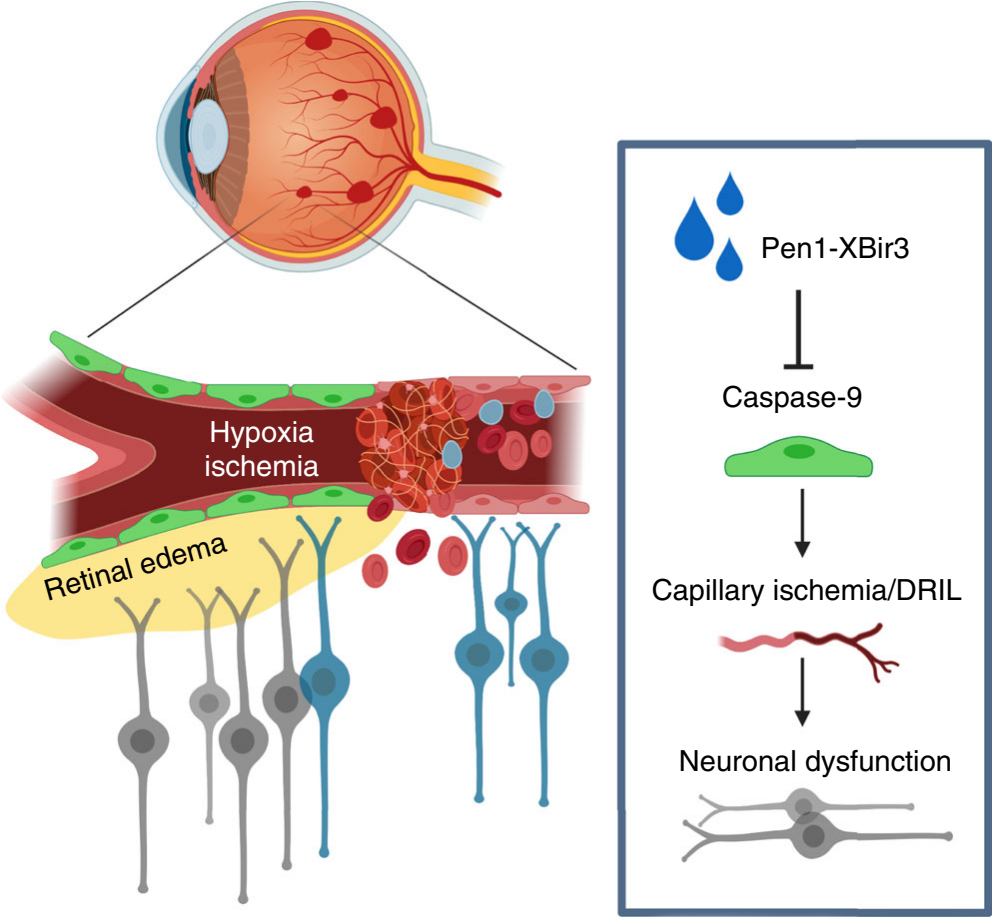
Model of caspase-9 activity in ischemic neurovascular injury. Hypoxia–ischemia injury activates caspase-9 in endothelial cells, inducing nonapoptotic endothelial dysfunction. Caspase-9 promotes capillary ischemia and induces endothelial barrier breakdown and neuronal injury. Pen1-XBir3 protects CNS tissues from hypoxia–ischemia injury by inhibiting caspase-9.

Vascular endothelium, which forms the blood brain/retinal barrier, is uniquely positioned as an active interface between neurons and inflammatory processes^60^. We report that in RVO, nonapoptotic activation of endothelial caspase-9 orchestrates vascular dysfunction and neuronal injury, supporting a non-cell autonomous consequence of endothelial caspase-9 activity (Fig. 9). RVO-induced caspase activation in neurons is driven by caspase-9 mediated endothelial dysfunction. We used inducible endothelial caspase-9 knockout mice to interrogate the endothelial cell specific role of caspase-9 in RVO pathology, and we also provide a tool, Pen1-XBir3, with which to investigate caspase-9 function via pharmacological approaches. Inhibition or genetic excision of caspase-9 from endothelial cells rescues vascular integrity and restores neuronal activity in an adult model of retinal hypoxia/ischemia. Taken together, these data suggest that targeting endothelial cell pathways could provide effective therapies for attenuating ischemic neuronal injury, and identify a nonapoptotic role for caspase-9 and caspase-7 in vascular barrier dysfunction.

Proteomics studies indicate that RVO elicits changes across a broad array of signaling pathways, highlighting the molecular complexity of hypoxia/ischemia injury^61^. The chronic nature of ischemia and microvascular dysfunction in human disease further adds to the complexity of retinal vascular disorders. Multiple causative pathways have been implicated in the pathogenesis of retinal edema, including VEGF signaling, the kinin/kallikrein system, and the Angiopoietin-2/Tie2 axis^1^. Our results identify nonapoptotic endothelial caspase-9 signaling as a key mediator of ischemic injury, and underscore the importance of vascular endothelial dysfunction in CNS hypoxia–ischemia injury. These results invite further exploration into the mechanisms regulating caspase signaling during chronic retinal ischemia, the downstream targets of endothelial caspase-9 that mediate vascular dysfunction, and the process by which these pathways intersect with other factors known to mediate retinal vascular disease.

Caspase-9 activity has been tightly associated with initiation of the intrinsic apoptosis pathway, and far less is known about caspase biology in non-apoptotic processes^42^. However, a growing body of literature has identified physiologic functions of caspase-9 in development and differentiation, which are independent of apoptosis: caspase-9 promotes muscle and cardiomyocyte differentiation and proliferation^62^, hematopoietic development^63^, and neuronal maturation and axonal pathfinding^64,65^. Although less is known about potential nondeath roles of caspases in endothelial cells, recent work has suggested a potential barrier protective role for endothelial caspase-3^66^. These studies highlight that caspases can be active in cells that are not dying, and that the physiological consequence of caspase activation depends on cellular context. However, we have limited understanding of the nonapoptotic effects of caspase-9 in disease because the activation of effector caspases in disease tissues is routinely interpreted as a hallmark of apoptotic cell death.

We see low basal levels of caspase activation present in uninjured retinas. Basal activation of caspase-3 in various cell types, caspase-6 in retinal astrocytes, and caspases-9/-7 in neuronal processes indicates cell-type specific regulation of physiological caspase activity in adult CNS tissues. We identify caspase-7 as the main effector caspase induced by caspase-9 in RVO, and the only effector caspase induced by RVO in endothelial cells. While it is possible that caspase-3 induction and/or apoptosis in endothelial cells could be evoked by more extreme ischemic injury (such as complete abrogation of retinal blood flow), we did not detect induction of caspase-3 cleavage or endothelial cell death in our injury model.

Our findings also provide a mechanistic insight into the function of endothelial caspase-9 in systemic disorders with vascular barrier dysfunction. In vitro studies have indicated that pan-caspase inhibition may modulate endothelial secretion of prothrombotic and proinflammatory factors by regulating exocytosis of Weibel-Palade bodies, suggesting a mechanistic link between caspase activation and endothelial inflammation^67^. While few studies have looked at caspase-9 expression specifically in patient endothelial cells, increased caspase-9 has been reported in atherosclerotic lesions^68^ and in Behcet’s disease, a multisystem disorder driven by endothelial dysfunction, which often includes ocular findings of retinal edema and atrophy^69^. The distinction between cell-death and non-cell-death caspase signaling is important for identifying potential therapeutic targets in disease. Our findings invite re-examination of apoptotic vs nonapoptotic caspase-9 pathways in disease pathologies. Taken together, these data suggest that endothelial caspase-9 may play a direct role in mediating endothelial barrier dysfunction and neuronal injury, and that targeting endothelial cell pathways could provide effective therapies towards supporting neuronal health.

## Methods

### Contact for reagent and resource sharing

Further information and requests for resources and reagents should be directed to and will be fulfilled by the Lead Contact, Carol M. Troy (cmt2@cumc.columbia.edu).

### Animals

Wild type C57Bl/6J 2-month-old mice were purchased from Jackson Laboratories. Endothelial specific caspase-9 inducible knockout (Casp9 iEC KO) mice were bred by crossing Caspase-9 flox/flox mice^57^ and endothelial cell-CreERT2 mice(Cdh5(PAC)-CreERT2)^58^. Cre reporter used mT/mG mice^59^. All mice are on a C57/Bl6 background. Recombination was induced in 6-week-old animals by intraperitoneal (IP) injection with 2 mg tamoxifen for 5 consecutive days. Male and female mice were used to characterize the knockout strains at baseline and in the RVO experiments on C57Bl/6J mice, and male Casp9 WT and Casp9 iEC KO mice were used in the RVO experiments.

An ocular distribution study of Pen1-XBir3 eye drops was performed by EyeCRO (Oklahoma City, OK). Adult female New Zealand White Rabbits had plasma collected and then received bilateral topical administration of 200 µg Pen1-XBir3, twice daily for a period of 4.5 consecutive days. On day 5, 4 h after administration of the final dose of Pen1-XBir3, plasma was collected from the animals, and the eyes were enucleated and the retinas dissected.

All investigations were performed in accordance with the ARVO statement for the Use of Animals in Ophthalmic and Vision Research. Rodent experiments were approved and monitored by the Institutional Animal Care and Use Committee (IACUC) of Columbia University, and the rabbit experiments were overseen by the IACUC of  EyeCRO.

### RVO model

RVO was performed by investigators blinded to genotype and treatment. Rose bengal (150 µl, 5 mg/mL) was administered to awake animals by tail vein injection 15 min prior to induction of RVO. Immediately prior to all imaging and RVO procedures, mice were anesthetized with ketamine (80–100 mg/kg) and xylazine (5–10 mg/kg). Eyes were dilated with tropicamide and phenylephrine chloride eye drops. All major veins (n = 3-6) in each eye were irradiated using Micron IV image guided laser (532 nm) (Phoenix Research Labs, Pleasanton, CA, USA). Three adjacent laser pulses (power 100 mW, spot size 50 µm, duration 1 s, 0.3 J total energy) were delivered to each vein an average distance of 375 µm from the optic nerve center. Laser application causes a vaporization bubble visible on fundus imaging, which covers <4% of total retinal area (Fig. 2a). Sham RVO was performed using identical parameters as the RVO procedure, with three adjacent laser burns targeted to the capillary bed between each of the major veins. RVO was judged successful if a vein occlusion persisted through 24 h post-RVO as observed by fundus imaging. Occluded veins were identified by a whitening of the vessel at the site of the occlusion, dilation distal to the occlusion, and constriction of vessel diameter proximal to the occlusion.

### Fluorescein angiography

Fluorescein angiography images were captured 24 h, 48 h, and 8 days post-RVO using a Micron IV fundus camera. Fluorescein visualization of vascular morphology in Casp9 iEC KO and Casp9 WT animals was performed 10 min after IP injection with 1% fluorescein. Fluorescein imaging of edema was performed 5 min after IP injection of 1% fluorescein. Fluorescein leakage was analyzed using Image J by measuring the mean fluorescein signal in the capillary bed around an occluded vein and normalized against the mean signal intensity of fluorescein within major arteries adjacent to the area measured.

### Image-guided OCT

OCT images were captured using the Phoenix Micron IV image-guided OCT system. For each eye, two vertical and two horizontal OCT scans were captured approximately 75 μm distal from the periphery of the RVO burn areas. For each eye, four OCT images were averaged to generate mean retinal thickness values. Segmentation of individual retinal layers was generated using InSight software, and average layer thicknesses were calculated in Excel. Specific retinal layers were identified as indicated in Fig. 1a. Total retinal thickness was measured from the GCL to the RPE, while intraretinal thickness was measured from GCL to the outer segment (OS). Retinal detachment was measured as distance from the OS to the RPE. Change in retinal thickness for injured eyes was measured against composite averages from uninjured controls.

HRF were analyzed from OCT images using Image J. OCT images were processed using Despeckle function. A threshold was applied to the INL, selecting hyperreflective regions as pixels 2 standard deviations above the mode INL pixel intensity. Number of HRF was quantified using Analyze Particles function.

DRIL was defined as the horizontal extent of each OCT image for which any boundaries between the IPL, INL, and OPL could not be identified. DRIL extent was measured using Image J.

### Focal ERG

Electroretinograms were recorded 7 days post-RVO with the Micron IV Image-Guided Focal ERG system on dark-adapted mice, using a flash spot size of 1.5 mm, centered on the optic nerve head (Supplementary Fig. 6F), and a 10 ms white light LED stimulus intensity of −0.7 log (Cd s/m^2^) and 2.3 log (Cd s/m^2^). Amplitudes of the a wave and b wave were calculated with Labscribe3 ERG. Oscillatory potentials (OP) were derived using a 30–300 Hz filter, and the sum of the first 6 OP was used to calculate sum OP amplitude.

### Immunohistochemistry and antibodies

All antibodies and dilutions for immunohistochemistry and Western blotting are listed in Supplement Table 1. Mice were euthanized with Ketamine 80–100 mg/kg plus Xylazine 5–10 mg/kg and perfused followed by fixation with 4% paraformaldehyde. Retinal flatmounts were permeabilized for 2 h at room temperature (RT) in PBS with 1% Triton X-100, and retinal sections were permeabilized in PBS with 0.1% Triton X-100, prior to blocking step. Retinal flatmounts and cross-sections were blocked with 10% normal goat serum/1% bovine serum albumin (BSA) in PBS, incubated with primary antibody overnight at 4 °C, washed with PBS, and incubated with the species-appropriate Alexa Fluor-conjugated secondary antibody (Invitrogen) for 2 h at RT. Sections and flatmounts were imaged using Zeiss LSM 800 and XlightV2 (Biovision Technologies) confocal microscopes, and the images processed in FIJI. Quantification was based on cell counts from 3-6 fields of view per tissue section, and an average of four sections per eye. Vascular staining was identified by morphology and colocalization with CD31 or isolectin. Neuronal staining was identified by nuclear and cell body morphology, localization with neuroretinal layers and absence of vascular markers.

### Western blot analysis

Western blots were blocked for 1 h with 5% BSA in TBS-Tween (0.05%), incubated with primary antibody overnight at 4 °C, washed with TBS-Tween, and incubated with the species-appropriate secondary antibodies for 2 h at RT. Western blots were imaged using a Licor Odyssey system. Western blots were quantified using Image Studio Lite software.

### Pen1-XBir3

His-tagged XBir3 and mutXBir3 (inactive mutant) were expressed in *Escherichia coli* and purified by nickel column. Pen1 (PolyPeptide Group) was mixed at a 2:1 molar ratio with purified XBir3 and incubated for 1–2 h at 37 °C to generate disulfide-linked Pen1-XBir3. Linkage was assessed by 20% sodium dodecyl sulfate polyacrylamide gel electrophoresis and Western blotting with anti-His antibody. Eye drops containing 10 µg Pen1-XBir3 were administered immediately following RVO, and again at 24 h (Fig. S5A). Equivalent volumes of saline containing unlinked Pen1, 10 µg unlinked XBir3, and 10 µg Pen1-mutXBir3 (inactive mutant) were administered as controls.

### Quantification and statistical analysis

RVO procedures and analyses were performed by investigators blinded to the treatment groups. In RVO studies, excessive damage, characterized by complete retinal detachment within 48 h post-RVO, or intravitreal hemorrhaging that obscured view of the retina, occurred in <15% of eyes, which were excluded by a blinded observer from all subsequent analyses. Eyes were included for subsequent analysis if fundus imaging identified occlusion of at least 1 vein sustained through 24 h post-RVO, and OCT analysis of retinal layer thickness was within 2.5 standard deviations of the mean for each treatment group. Data were analyzed using Excel and GraphPad Statistical software. Statistical tests, *n* values and *p* values are all located in the figures and/or legends. Significance was defined as *p* < 0.05. No statistical methods were used to predetermine sample size. Target sample size for in vivo imaging data in RVO experiments was determined during the establishment of the RVO model based on the variability of the OCT thickness measurements.

### Reporting summary

Further information on research design is available in the Nature Research Reporting Summary linked to this article.

## Supplementary information

Supplementary Information

Reporting Summary

## Data Availability

Raw data is available on reasonable request. The source data for Figs. 1–8 is provided in the Source Data file Source data are provided with this paper.

## Source data

Source Data
