# Author Correction: Endothelial activation of caspase-9 promotes neurovascular injury in retinal vein occlusion

**DOI:** 10.1038/s41467-020-17476-y

**Published:** 2020-07-09

**Authors:** Maria I. Avrutsky, Crystal Colón Ortiz, Kendra V. Johnson, Anna M. Potenski, Claire W. Chen, Jacqueline M. Lawson, Alexandra J. White, Stephanie K. Yuen, Fatima N. Morales, Elisa Canepa, Scott Snipas, Guy S. Salvesen, Ying Y. Jean, Carol M. Troy

**Affiliations:** 1 Department of Pathology & Cell Biology; Vagelos College of Physicians and Surgeons, Columbia University, New York, NY 10032 USA; 2 Department of Pharmacology; Vagelos College of Physicians and Surgeons, Columbia University, New York, NY 10032 USA; 3 NCI-designated Cancer Center, Sanford Burnham Prebys Medical Discovery Institute La Jolla, La Jolla, CA 92037 USA; 4 Department of Neurology; Vagelos College of Physicians and Surgeons, Columbia University, New York, NY 10032 USA; 5 The Taub Institute for Research on Alzheimer’s Disease and the Aging Brain; Vagelos College of Physicians and Surgeons, Columbia University, New York, NY 10032 USA

**Keywords:** Cell biology, Cell death, Mechanisms of disease, Neuroscience, Pathogenesis

Correction to: *Nature Communications* 10.1038/s41467-020-16902-5, published online 23 June 2020.

The original version of this Article contained an error in the author affiliation.

Guy S. Salvesen was incorrectly associated with Department of Pharmacology; Vagelos College of Physicians and Surgeons, Columbia University, New York, NY 10032, USA.

This has now been corrected in both the PDF and HTML versions of the Article.

