# Supplementary material for: Endothelial activation of caspase-9 promotes neurovascular injury in retinal vein occlusion
Source: Nat Commun. 2020 Jun 23;11:3173. doi: 10.1038/s41467-020-16902-5 (PMC7311551; doi:10.1038/s41467-020-16902-5)
Supplement: Supplementary file 1 — Supplementary Information [file 41467_2020_16902_MOESM1_ESM.pdf]

Endothelial activation of caspase-9 promotes neurovascular injury in retinal vein occlusion

M.I. Avrutsky et al.

Supplementary Figures and Information

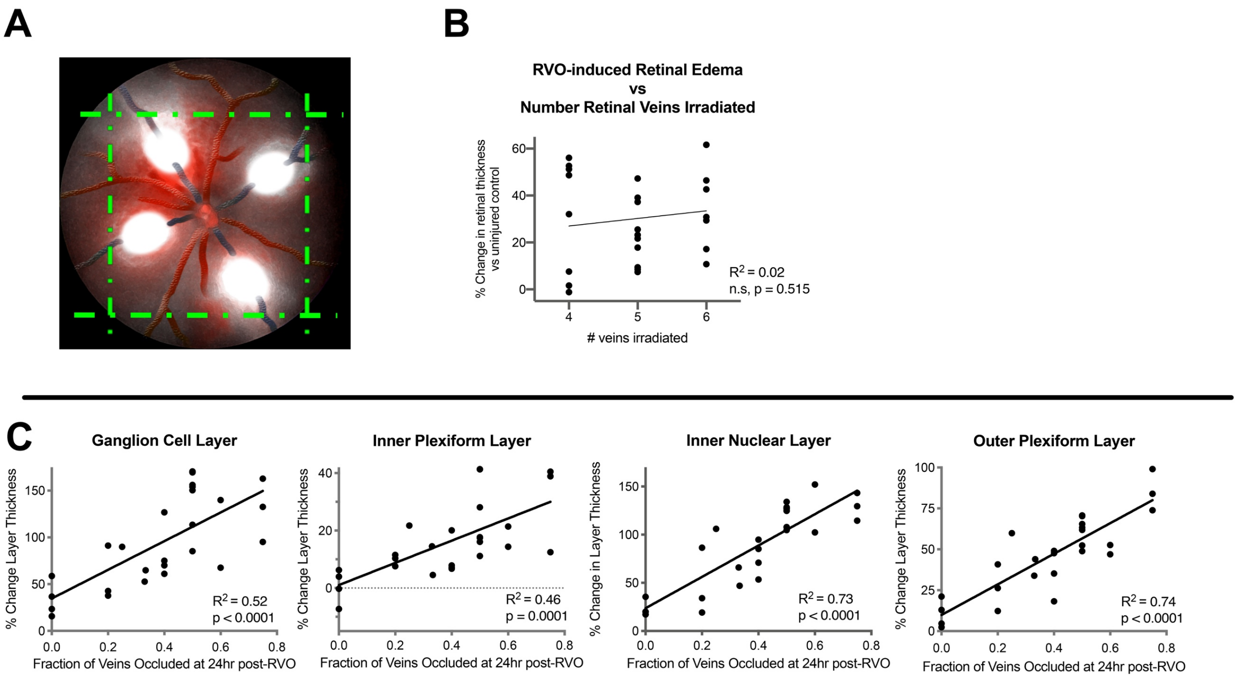

Supplement Figure 1. Related to Figure 1. Correlation of retinal vein occlusion and swelling of retinal layers

- **A.** Schematic illustration of where OCT measures are acquired relative to positioning of laser burns. Average OCT retinal thickness is derived from the average of 4 OCT images acquired distal from laser burn areas, as indicated by green dashed lines.
- **B.** RVO was induced by irradiating all major retinal veins (4-6). Higher laser burden in eyes with 6 irradiated veins does not correlate with greater edema (linear regression, n=25).
- **C.** Retinal swelling in individual retinal layers is correlated to fraction of veins occluded at 24hr post-RVO (linear regression, n=25).
  Source data are provided as a Source Data file.

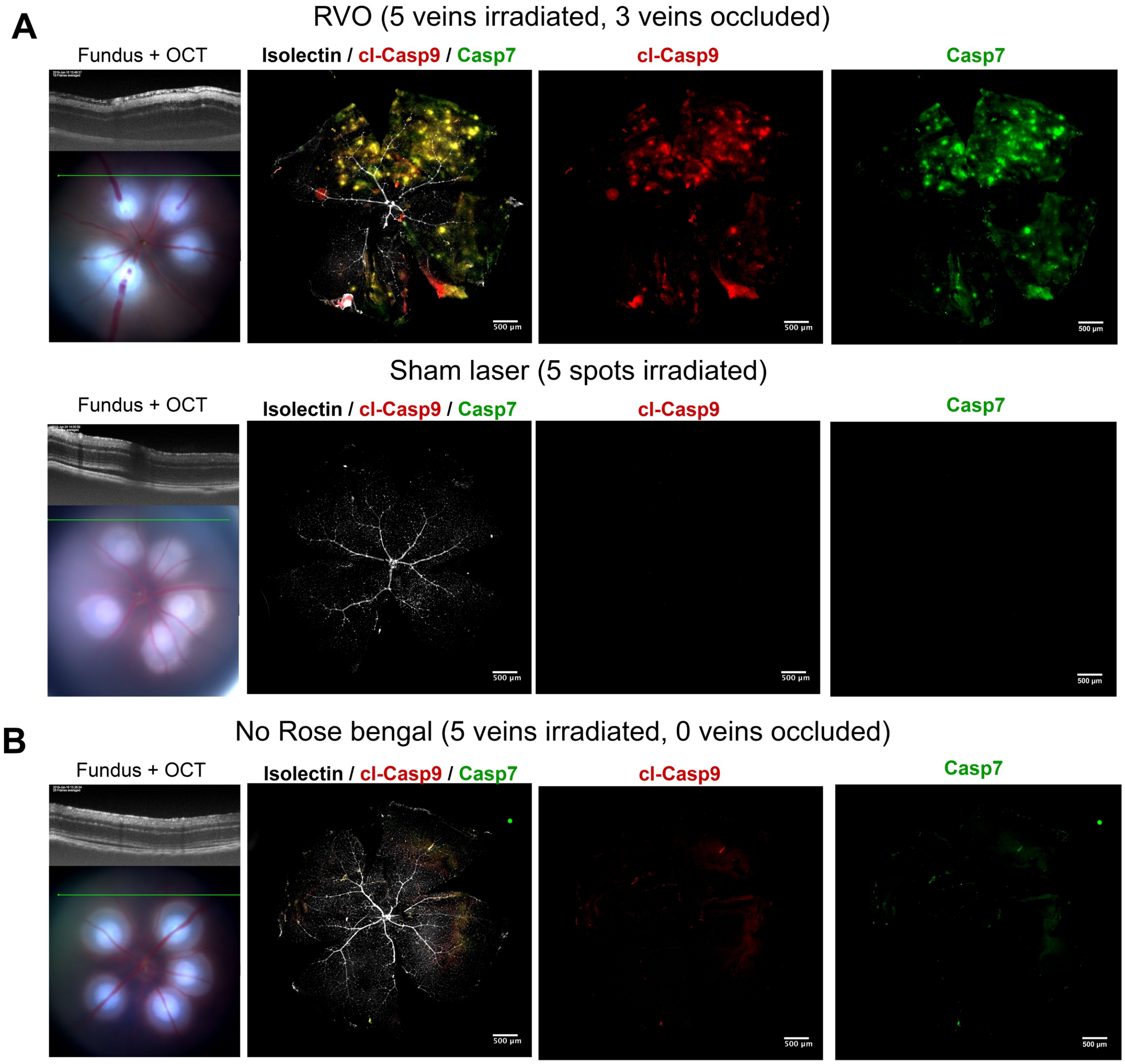

Supplement Figure 2. Related to Figure 2. Laser irradiation does not induce retinal edema or caspase-9 signaling

- **A.** *In vivo* imaging and immunostaining of retinas 4 hours post-RVO or sham laser (white=isolectin, red=cl-Casp9, green=Casp7, n=4, scale bar = 500μm).
- **B.** *In vivo* imaging and immunostaining of retinas 4 hours post application of laser burns to veins without occlusion. Veins were irradiated using standard laser settings, but without administration of Rose Bengal, resulting in no occlusions. (white=isolectin, red=cl-Casp9, green=Casp7, n=4, scale bar = 500μm). cl-casp9, cl-caspase-9; casp7, caspase-7. Source data are provided as a Source Data file.

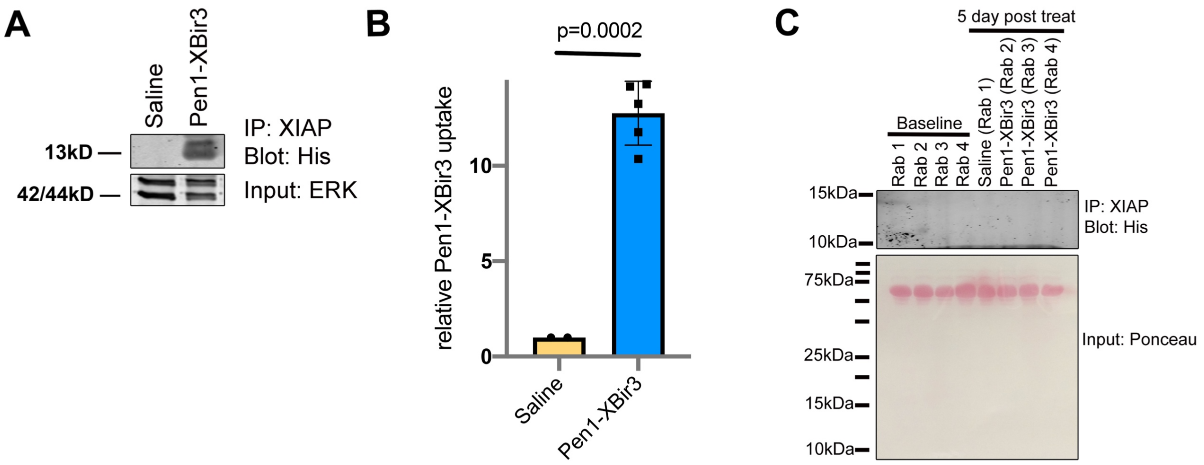

Supplement Figure 3. Related to Figure 3. Eye-drop delivery of Pen1-XBir3 to rabbit eyes

- **A.** Western blot showing detection of XBir3 in retinal lysates following administration of Pen1-XBir3 eye-drops in rabbits for 5 consecutive days.
- **B.** Quantification; two-tailed unpaired t-test, n=2 retinas (saline) n=5 retinas (Pen1-XBir3); mean ±SEM
- **C.** Western blot showing no detection of XBir3 in rabbit plasma after administration of Pen1-XBir3 eye-drops for 5 consecutive days. n=1 rabbit (saline), n=3 rabbits (Pen1-XBir3).
  Source data are provided as a Source Data file.

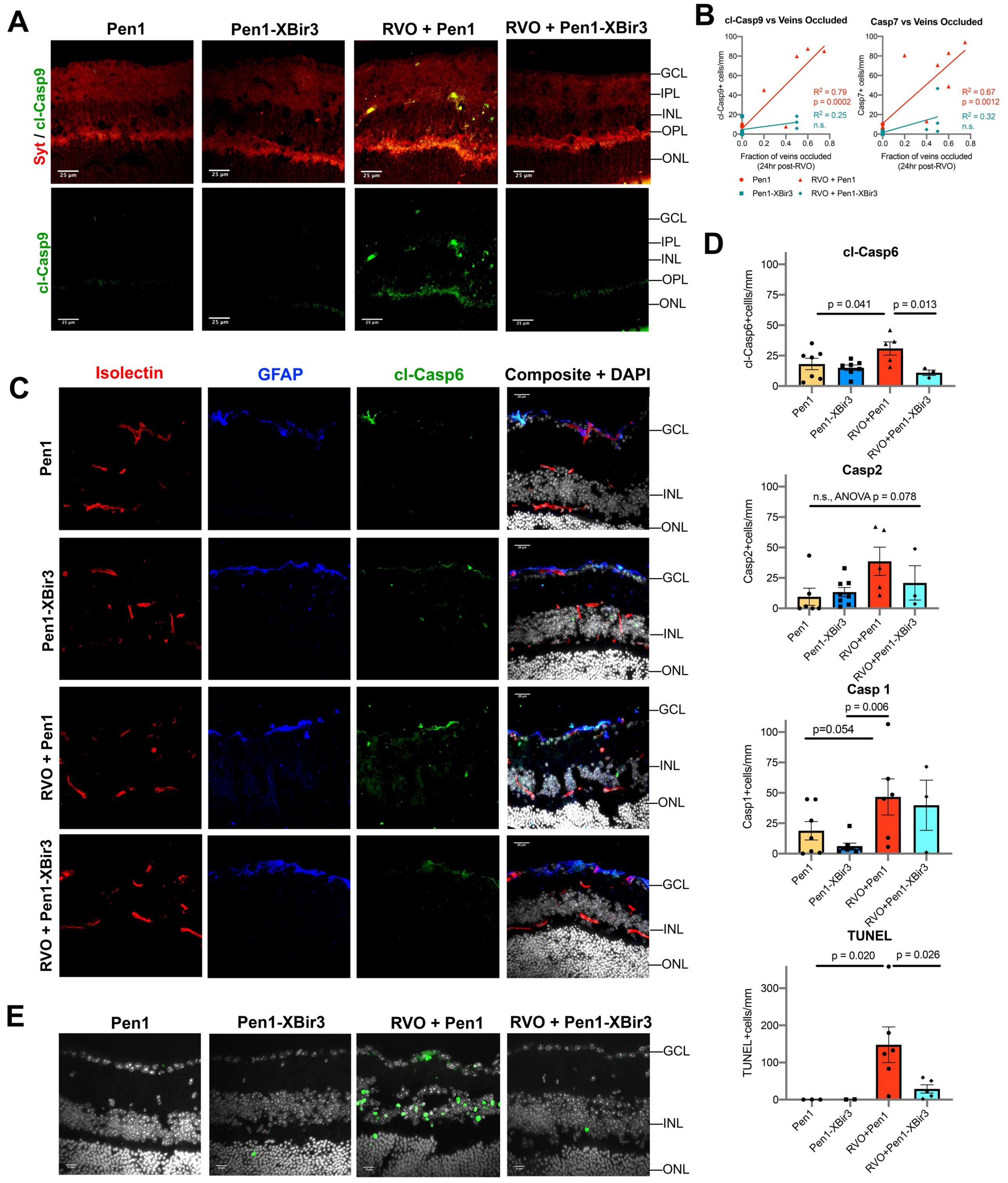

Supplement Figure 4. Related to Figure 4. Pen1-XBir3 inhibits caspase-9 signaling post-RVO

Supplement Figure 4. Related to Figure 4. Pen1-XBir3 inhibits caspase-9 signaling post-RVO

A. Synaptotagmin (red) marks neuronal processes in the IPL and OPL. Low levels of cl-Casp9 (green) colocalize with synaptotagmin in uninjured eyes. Expression of cl-Casp9 in neuronal processes increases 24hr post-RVO, and is blocked by Pen1-XBir3. (scale bar = 25μm, n=3).

B. Induction of cl-Casp9 and cl-Casp7 is correlated to fraction of veins occluded in Pen1 and not in Pen1-XBir3-treated eyes (Linear regression, cl-Casp9; Pen1 (n=6), RVO + Pen1 (n=5), Pen1-XBir3 (n=5), RVO + Pen1-XBir3 (n=3), clCasp7; Pen1 (n=6), RVO + Pen1 (n=6), Pen1-XBir3 (n=6), RVO + Pen1-XBir3 (n=4))

C. Retinal sections from control eyes and 24hr post-RVO, immunostained for isolectin (red), GFAP (blue), cl-Casp6 (green), and DAPI (white) (scale bar = 25μm). Pen1 (n=7), RVO + Pen1 (n=5), Pen1-XBir3 (n=7), RVO + Pen1-XBir3 (n=3)

D. Quantification of cl-Casp6, Casp 2 and Casp1 signal in control eyes and 24hr post-RVO; n=3-8, ANOVA with Fisher's LSD, mean ±SEM. Pen1 (n=6), RVO + Pen1 (n=5), Pen1-XBir3 (n=8), RVO + Pen1-XBir3 (n=3)

E. Retinal sections from control eyes and 24hr post-RVO, stained for TUNEL (green) and DAPI (white). (scale bar = 25μm). Quantification on right, one-way ANOVA with Fisher's LSD, mean ±SEM. Pen1 (n=3), RVO + Pen1 (n=6), Pen1-XBir3 (n=2), RVO + Pen1-XBir3 (n=5)
cl-casp9, cl-caspase-9; casp7, caspase-7; cl-casp6, cl-caspase-6; casp2, caspase-2, casp1, caspase1. Source data are provided as a Source Data file.

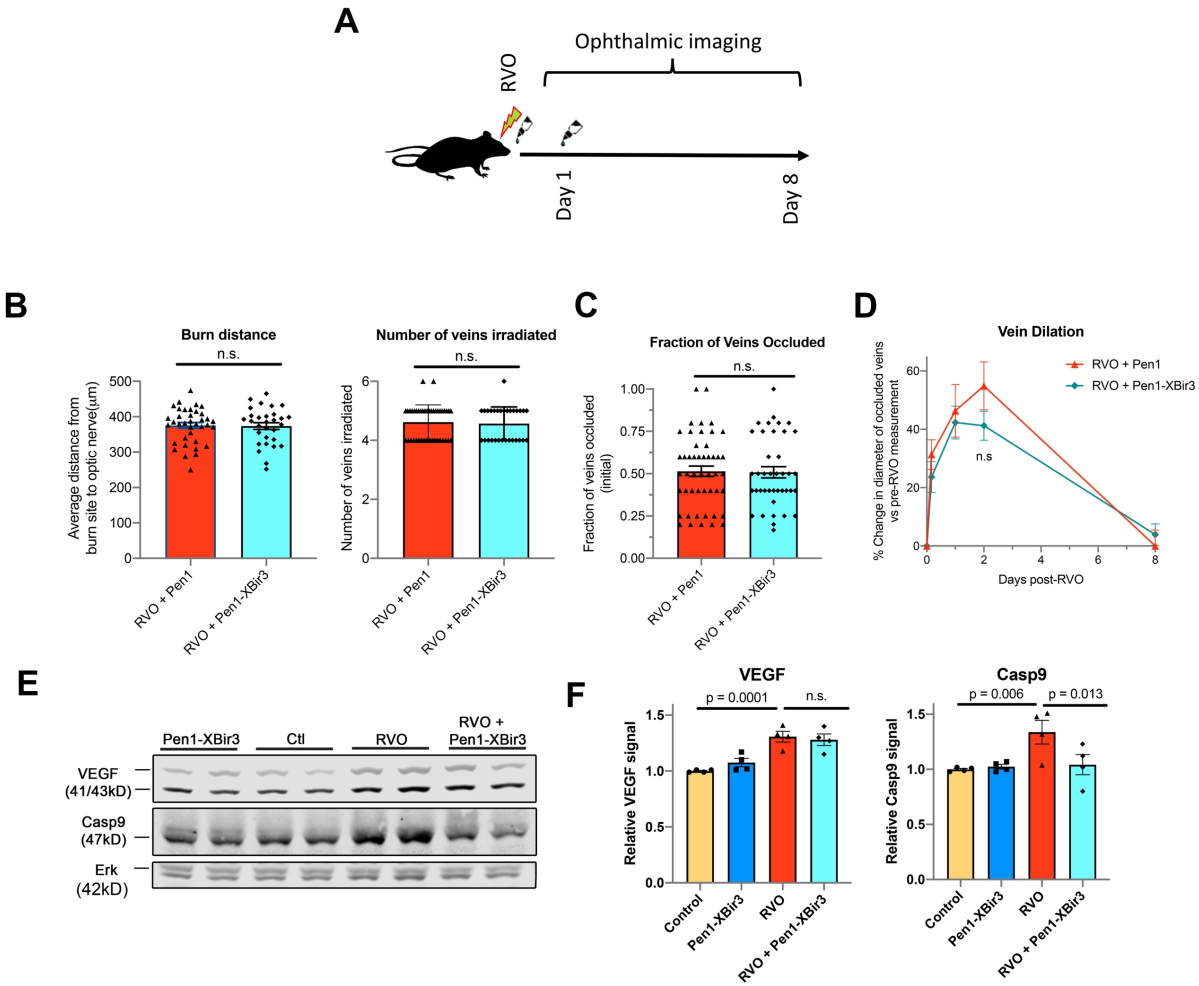

Supplement Figure 5. Related to Figure 5. Induction of RVO in Pen1 and Pen1-XBir3 treated eyes

- **A.** Schematic diagram of Pen1-XBir3 or Pen1 eye-drop administration in RVO studies; animals received first dose of eye-drops immediately after induction of RVO, and a second dose at 24hr post-RVO. Animals were followed through 8 days by *in vivo* ophthalmic imaging.
- **B.** Measurement of average laser-burn distance to optic nerve and number of veins irradiated in Pen1 (n=37) and Pen1-XBir3 (n=30) treated eyes; two-tailed Welch's t-test; mean ±SEM.
- **C.** Fraction of veins initially occluded after induction of RVO in Pen1 (n=37) and Pen1-XBir3 (n=30) treated eyes; two-tailed Welch's t-test; mean ±SEM.
- **D.** Measurement of changes in vein diameter in occluded veins in Pen1 (n=16, 11, 14, 13) and Pen1-XBir3 (n=11, 12, 16, 15) treated eyes at 4hr, 24hr, 48hr, and 8 days post-RVO; two-tailed Welch's t-test; mean ±SEM.
- **E.** Western blot of retinal lysates at 24hr post-RVO. Pen1-XBir3 eye-drops prevent induction of caspase-9 but not induction of VEGF.
- **F.** Quantification of E; one way ANOVA with Fisher's LSD, n=4; mean ±SEM.

Casp9, caspase-9. Source data are provided as a Source Data file.

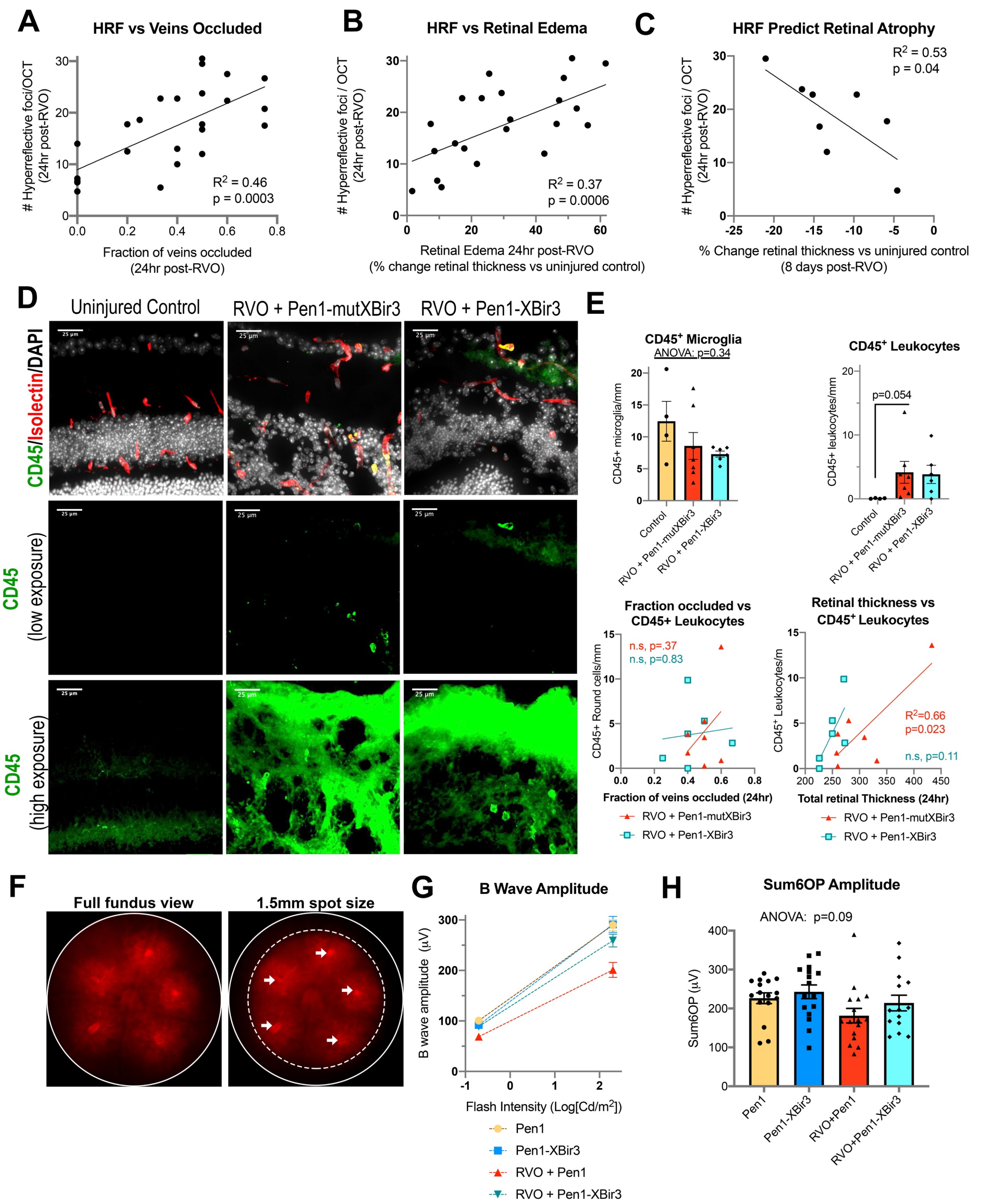

Supplement Figure 6. Related to Figure 6. HRF and ERG measurements following RVO

Supplement Figure 6. Related to Figure 6. HRF and ERG measurements following RVO

- **A.** Number of retinal HRF is correlated to fraction of veins occluded at 24hr post-RVO (linear regression, n=25)
- **B.** Number of retinal HRF is corelated to retinal thickness 24hr post-RVO (linear regression, n=25)
- **C.** Number of retinal HRF at 24hr post-RVO is correlated to retinal thickness at 8 days post-RVO (linear regression, n=8)
- **D.** Retinal sections from control eyes (n=4) and 24hr post-RVO treated with Pen-XBir3 (n=6) and Pen1-mutXBir3 (n=7) stained with CD45 (green), Isolectin (red) and DAPI (white). CD45 depicted at two brightness intensities. Scale bar = 25µm
- **E.** Quantification of images in D, showing number of CD45+ microglia and CD45+ leukocytes (one-way Welch's ANOVA with unpaired Welch's t-test, mean ±SEM), and correlation between CD45+ leukocytes and fraction of veins occluded and retinal edema 24hr post-RVO (linear regression).
- **F.** Illustration of spot size parameters for focal ERG measurements. Full retinal fundus view indicated via solid circle; retinal illumination by 1.5mm spot size centered around optic nerve indicated by dashed circle. Laser burns indicated with arrows.
- **G.** Quantification of b wave amplitude in uninjured control (n=16) and 7 days post-RVO (n=15) in Pen1 and Pen1-XBir3 treated eyes; mean ±SEM.
- **H.** Quantification of sum oscillatory potentials (OP) amplitude in uninjured control eyes (n=16) and 7 days post-RVO in Pen1 (n=16) and Pen1-XBir3 (n=14) treated eyes; One-way ANOVA; mean ±SEM.

Source data are provided as a Source Data file.

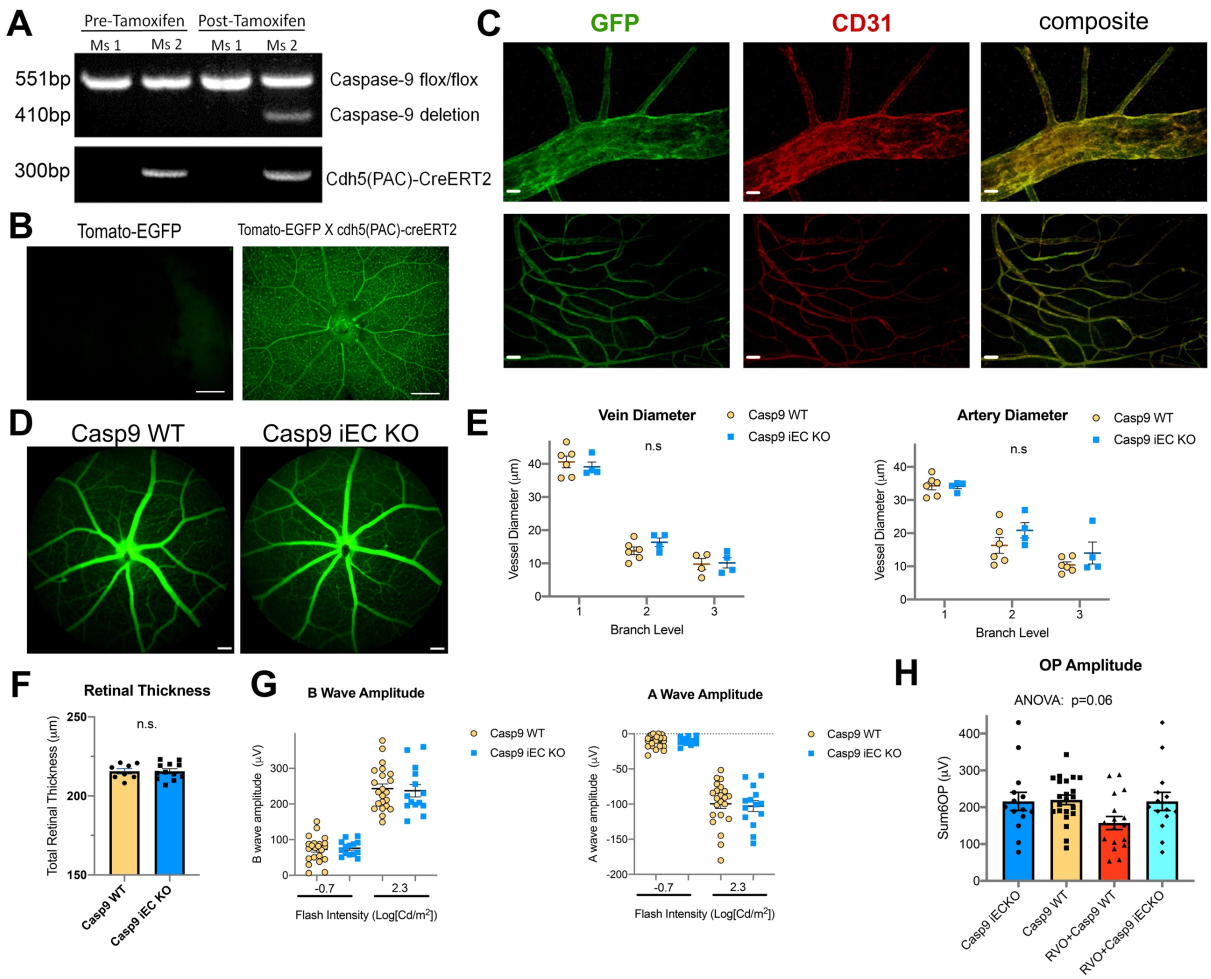

Supplement Figure 7. Related to Figure 7. Characterization of inducible endothelial caspase-9 knockout (Casp9 iEC KO) mice

A. Genotyping PCR of Casp9 WT mouse (Ms1) and Casp9 iEC KO mouse (Ms2) before and after tamoxifen treatment shows induction of caspase-9 deletion in Ms2; n=2

B. GFP fluorescence in retinal flatmount of Tomato-EGFP reporter mouse and Tomato-EGFP Cdh5(PAC)-CreERT2 mouse following tamoxifen induction of recombination; n=2; scale bar=500µm

C. Flatmount of EGFP X Cdh5(PAC)-CreERT2 retina following tamoxifen induction, immunostained with GFP and endothelial marker CD31 shows recombination in large vessels (top) and microvasculature (bottom) (scale bar=29µm).

D. Fluorescein angiography of female Casp9 WT and Casp9 iEC KO mice show no differences in retinal vascular morphology or retinal vascular permeability. scale bar=100µm

E. Quantification of vein and artery diameters from fluorescein angiography images of Casp9 WT (n=6) and Casp9 iEC KO (n=4) mice; two-tailed Welch's t-test; mean ±SEM.

F. Quantification of baseline OCT retinal thickness in Casp9 WT (n=8) and Casp9 iEC KO (n=12) eyes; Welch's t-test; mean ±SEM

G. Quantification of scotopic ERG b-wave amplitude and a-wave amplitude, in Casp9 WT (n=22) and Casp9 iEC KO (n=11) eyes under dim (-0.7log(Cd/m^2)) and bright (2.3log(Cd/m^2)) flash stimulus shows no difference in basal ERG response; mean ±SEM

H. Quantification of sum oscillatory potential (OP) amplitude 7 days post-RVO in Casp9 WT (uninjured n=22, RVO n=17) and Casp9 iEC KO eyes (uninjured n=14, RVO n=14) (flash intensity = 2.3log(Cd/m^2); One-way ANOVA; mean ±SEM.

Source data are provided as a Source Data file.

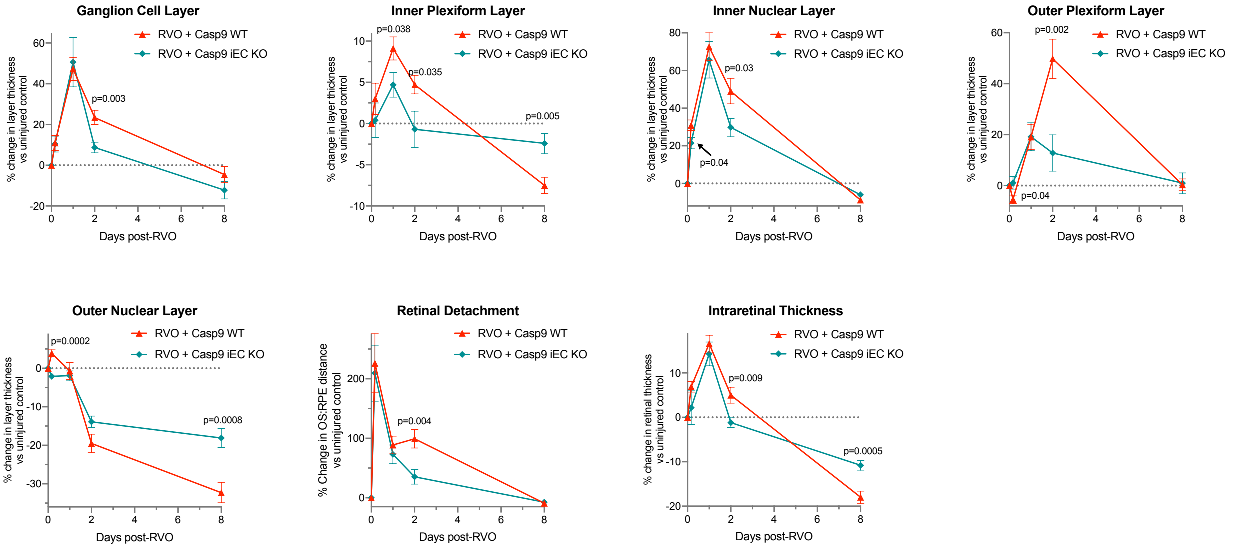

Supplement Figure 8. Related to Figure 8. Layer-specific changes in OCT retinal thickness
Quantification of changes in average OCT retinal thickness in specific retinal layers relative to baseline thickness of uninjured controls. RVO + Casp9 WT (n=12, 22, 12, 10), RVO + Casp9 iEC KO (n=7, 19, 11, 13); two-tailed Welch's t-test; as mean ±SEM. Source data are provided as a Source Data file.

Table 1 (Key Resources)

Antibodies				
Target	Supplier	Catalog Number	Application (validation)	Dilution
Caspase-9	Abcam	Ab28131	WB (validated by supplier for WB)	1:1000
cl-Caspase-9	Cell Signaling	9505-S	IHC (validated by supplier for WB, IP in human tissue)	1:800
cl-Caspase-9	Abcam	Ab2325	IHC, WB (validated by supplier for WB, ICC)	IHC 1:50<br>WB: 1:500
Caspase-7 488	Novus Biologicals	NB-56529AF488	IHC (validated by supplier for IHC)	1:150
cl-Caspase-3 647	Cell Signaling	#9602	IHC (validated by supplier for IF-IC)	1:50
Caspase-8	Cell Signaling	#4790S	IHC (validated by supplier for WB)	1:100
cl-Caspase-6	Cell Signaling	#9761	IHC (validated by supplier for WB)	1:100
Caspase-2	Enzo	ALX 804-355-C100	IHC (validated by supplier for ICC)	1:100
Caspase-1 488	Novus	NB100-S56565	IHC (validated by supplier for ICC/IF)	1:100
CD45 (D3F8Q)	Cell Signaling	70257S	IHC (validated by supplier for IHC)	1:100
CD31	BD Pharmigen	553370	IHC (validated by supplier for IHC)	1:50
GFP	Life Tech	A11122	IHC (validated by supplier for IHC)	1:1000
GFAP	Sigma	G-3893	IHC (validated by supplier for IHC)	1:2000
Isolectin (594, 649)	Vector	DL-1207	IHC (validated by supplier for IF)	1:200
XIAP	Cell Signaling	2042	IP (validated by supplier by WB)	1:200
ERK	Santa Cruz	SC-93	WB (validated by supplier for WB)	1:5000
His	GenScript	A00186	WB (validated by supplier for WB)	1:5000
VEGF	Abcam	Ab46154	WB (validated by supplier for WB)	1:1000
Synaptotagmin	Aves	STG	IHC (validated by supplier for IHC)	1:2000

Chemicals, Peptides, and Recombinant Proteins	
Penetratin -1 (Pen1):<br>C(NPys)-RQIKIWFQNRRMKWKK	PolyPeptide Group
XBir3:<br>MGSSHHHHHHSSGLVPRGSHMSTNLPRNPSMADYEARIFTFGTWIYSVNKEQLARAGFYALGEGDKVKCFHCGGGLTDWKPSEDPWEQHAKWYPGCKYLLEQKGQEYINNIHLTHS	Salvesen lab

XBir3[Q319R, W323V] (inactive mutant): MGSSHHHHHHSSGLVPRGSHMSTNLPRNPSMADYEARIFTFGTWIYSVNKEQLARAGFYALGEGDKVKCFHCGGGLTDWKPSEDPWE**R**HAK**V**YPGCKYLLEQKGQEYINNIHLTHS		Salvesen lab
Tamoxifen	Sigma	330000
Rose bengal	Sigma	1002604051
Ketamine	Henry Schein	
Xylazine	Henry Schein	139-236
Tropicamide	Akorn	NDC 17487-102-12
Phenylephrine chloride	Akorn	NDC 17478-201-15
Proparacaine	Akorn	NDC 17478-263-12
Fluorescein	Akorn	NDC 17478-253-10
Carprofen	Henry Schein	NDC 11695-6934-1
DeadEnd Fluorometric TUNEL System	Promega	G3250
Experimental Models: Organisms/Strains		
C57/Bl6J	Jackson Labs	Stock # 00664
Caspase-9 flox/flox	Genentech	(Simon et al., 2012)
Endothelial cell-CreERT2 (Cdh5(PAC)-CreERT2)	R. Adams	(Pitulescu et al., 2010)
Cre Reporter mT/mG	Jackson Labs	Stock # 007676
Adult female New Zealand White Rabbits	EyeCro	
Oligonucleotides		
Primers for caspase-9 genotyping:<br>1: GCAACTTGGAACCCACATG<br>2: GGACAGATGGATGCTCCGT<br>3: GGACCATTACCTCTACCTGCCT		Life Technologies
Primers for Cdh5(PAC)-CreERT2 genotyping:<br>1: GATATCTCACGTACTGACGG<br>2: TGACCAGAGTCATCCTTAGC		Life Technologies
Software and Algorithms		
Insight 2D Version 2	Phoenix Research Labs	
Labscribe3 ERG 3.0168	Phoenix Research Labs	
Fundus retinal imaging: StreamPix version 6	Phoenix Research Labs	

MicronOCT version 7.2.4.2	Phoenix Research Labs	
Confocal imaging: ZEN 2.3 (blue edition)	ZEISS	
Confocal imaging: VisiView 4.0	Visitron Systems	
Western Imaging: Licor Image Studio 5.x	Li-Cor	
Western blot analysis: Licor Image Studio Lite 5.2.5	Li-Cor	
FIJI 1.0		
Prism 8.3.1	GraphPad	
Excel Version 16.27	Microsoft	
Other		
Micron IV retinal imaging system	Phoenix Research Labs	
