# Supplementary material for: Endothelial activation of caspase-9 promotes neurovascular injury in retinal vein occlusion
Source: Nat Commun. 2020 Jun 23;11:3173. doi: 10.1038/s41467-020-16902-5 (PMC7311551; doi:10.1038/s41467-020-16902-5)
Supplement: Supplementary file 2 — Reporting Summary [file 41467_2020_16902_MOESM2_ESM.pdf]

nature research

Corresponding author(s): Carol M. Troy

Last updated by author(s): April 21, 2020

# Reporting Summary

Nature Research wishes to improve the reproducibility of the work that we publish. This form provides structure for consistency and transparency in reporting. For further information on Nature Research policies, see Authors & Referees and the Editorial Policy Checklist .

## Statistics

For all statistical analyses, confirm that the following items are present in the figure legend, table legend, main text, or Methods section.

| n/a | Confirmed | |
|---|---|---|
| ☐ | ☒ | The exact sample size (*n*) for each experimental group/condition, given as a discrete number and unit of measurement |
| ☒ | ☐ | A statement on whether measurements were taken from distinct samples or whether the same sample was measured repeatedly |
| ☐ | ☒ | The statistical test(s) used AND whether they are one- or two-sided<br>*Only common tests should be described solely by name; describe more complex techniques in the Methods section.* |
| ☐ | ☒ | A description of all covariates tested |
| ☐ | ☒ | A description of any assumptions or corrections, such as tests of normality and adjustment for multiple comparisons |
| ☐ | ☒ | A full description of the statistical parameters including central tendency (e.g. means) or other basic estimates (e.g. regression coefficient) AND variation (e.g. standard deviation) or associated estimates of uncertainty (e.g. confidence intervals) |
| ☐ | ☒ | For null hypothesis testing, the test statistic (e.g. *F*, *t*, *r*) with confidence intervals, effect sizes, degrees of freedom and *P* value noted<br>*Give P values as exact values whenever suitable.* |
| ☒ | ☐ | For Bayesian analysis, information on the choice of priors and Markov chain Monte Carlo settings |
| ☒ | ☐ | For hierarchical and complex designs, identification of the appropriate level for tests and full reporting of outcomes |
| ☐ | ☒ | Estimates of effect sizes (e.g. Cohen's *d*, Pearson's *r*), indicating how they were calculated |

*Our web collection on statistics for biologists contains articles on many of the points above.*

## Software and code

Policy information about availability of computer code

| | |
|---|---|
| Data collection | All retinal imaging data was collected using software included with the Phoenix Micron IV retinal imaging system (Labscribe ERG version 3, StreamPix version 6, MicronOct version 7, Insight 2D version 2)<br>Confocal Imaging acquired with ZEN 2.3 (blue edition, ZEISS) and VisiView 4.0 (Visitron Systems)<br>Western Blot imaging acquired with Licor Image Studio version 5 |
| Data analysis | Western blot analysis: Image studio lite version 5<br>OCT analysis: Insight 2D version 2<br>Image analysis: FIJI version 1<br>Statistics: Prism version 8, Excel version 16 |

For manuscripts utilizing custom algorithms or software that are central to the research but not yet described in published literature, software must be made available to editors/reviewers. We strongly encourage code deposition in a community repository (e.g. GitHub). See the Nature Research guidelines for submitting code & software for further information.

## Data

Policy information about availability of data

All manuscripts must include a data availability statement. This statement should provide the following information, where applicable:

- Accession codes, unique identifiers, or web links for publicly available datasets
- A list of figures that have associated raw data
- A description of any restrictions on data availability

The data that support the findings of this study are available from the corresponding author upon reasonable request. The source data for all figures and supplementary figures are provided with the manuscript.

# Field-specific reporting

Please select the one below that is the best fit for your research. If you are not sure, read the appropriate sections before making your selection.

☒ Life sciences ☐ Behavioural & social sciences ☐ Ecological, evolutionary & environmental sciences

For a reference copy of the document with all sections, see nature.com/documents/nr-reporting-summary-flat.pdf

# Life sciences study design

All studies must disclose on these points even when the disclosure is negative.

| | |
|---|---|
| Sample size | No statistical methods were used to predetermine sample size. Target sample size of n=16 eyes for in vivo imaging data in RVO experiments was determined during the establishment of the RVO model based on the variability of the OCT thickness measurements. For IHC and WB experiments, sample size was based on previous experience with these techniques. |
| Data exclusions | Eyes were excluded from further analysis if no vessel occlusion was achieved, or if ocular damage impaired in vivo retinal imaging, characterized by corneal cataract, complete retinal detachment within 48hr post-RVO, or intravitreal hemorrhaging that obscured view of the retina as determined by a blinded observer. Due to the inherent wide range of variability in retinal pathology post-RVO, an objective exclusion criteria was designed based on OCT analysis of retinal thickness: eyes were included for subsequent analysis if retinal thickness was within 2.5 standard deviations of the mean for each treatment group. ERG analysis excluded eyes were b-wave amplitude was >450uV at flash intensity of 2.3log(cd sec/m2) based on similar experiments showing outliers with exceptionally high b-wave readings. |
| Replication | These experiments are the subject of continuing work in our lab, and different aspects of the work have been reproduced in 3 independent projects in the lab. |
| Randomization | Treatment groups were randomly assigned to the mice prior to injury. |
| Blinding | Investigators were blinded to treatment group allocation, data was anonymized for analysis. |

# Reporting for specific materials, systems and methods

We require information from authors about some types of materials, experimental systems and methods used in many studies. Here, indicate whether each material, system or method listed is relevant to your study. If you are not sure if a list item applies to your research, read the appropriate section before selecting a response.

## Materials & experimental systems

| n/a | Involved in the study | |
|---|---|---|
| ☐ | ☒ | Antibodies |
| ☒ | ☐ | Eukaryotic cell lines |
| ☒ | ☐ | Palaeontology |
| ☐ | ☒ | Animals and other organisms |
| ☒ | ☐ | Human research participants |
| ☒ | ☐ | Clinical data |

## Methods

| n/a | Involved in the study | |
|---|---|---|
| ☒ | ☐ | ChIP-seq |
| ☒ | ☐ | Flow cytometry |
| ☒ | ☐ | MRI-based neuroimaging |

## Antibodies

| | |
|---|---|
| Antibodies used | Catalog information and dilutions for all antibodies used is provided in Supplement Table 1 |
| Validation | All antibodies used are commercially validated by the supplier; specific application validations are listed in Supplement Table 1. Additionally, IHC for cleaved-caspase-9, active caspase-7, and cleaved caspase-3 was independently confirmed using two different commercially available antibodies. |

## Animals and other organisms

Policy information about studies involving animals; ARRIVE guidelines recommended for reporting animal research

| | |
|---|---|
| Laboratory animals | Detailed references on animals are provided in the manuscript. Adult mice (2-4 months old) were used in the following strains: C57/Bl6J, caspase-9 flox, Cdh5(PAC)-CreERT2, mT/mG. Rabbit study: 3-4 month old female New Zealand White Rabbits. |
| Wild animals | No wild animals were used in the study. |
| Field-collected samples | No field-collected animals were used in the study. |

| | |
|---|---|
| Ethics oversight | All rodent procedures were approved by the Columbia University Institutional Animal Care and Use Committee and performed in accordance with the ARVO statement for the Use of Animals in Ophthalmic and Vision Research. |

Note that full information on the approval of the study protocol must also be provided in the manuscript.
